# Joint disease in systemic lupus erythematosus: clinical manifestations, immunopathology, and treatment

**DOI:** 10.3389/fimmu.2026.1959167

**Published:** 2026-10-16

**Authors:** Melissa Ong, Fareeha Tariq, Arthi Mahto, Chris Wincup

**Affiliations:** 1 Department of Rheumatology, West Hertfordshire Teaching Hospitals National Health Service (NHS) Trust, Watford, United Kingdom; 2 Department of Rheumatology, King’s College Hospital, London, United Kingdom; 3 Centre for Rheumatic Diseases, King’s College London, London, United Kingdom

**Keywords:** Jaccoud’s arthropathy, lupus arthritis, magnetic resonance imaging, musculoskeletal ultrasound, outcome measures, rhupus, subclinical synovitis, systemic lupus erythematosus

## Abstract

Joint involvement affects the majority of patients with systemic lupus erythematosus and is consistently ranked among the manifestations with the largest impact on daily function, yet lupus synovium remains one of the least interrogated major target tissues in this disease. The primary literature on lupus synovial tissue rests mainly on two datasets, neither generated by single-cell transcriptomic or mass cytometric methods, and what they describe is a relatively pauci-immune and myeloid-dominated compartment carrying an interferon-inducible transcriptional signature *in situ*, without the ectopic germinal centers and expanded pathogenic fibroblast populations that characterize rheumatoid synovitis. The cell populations now central to systemic lupus biology, among them T peripheral helper cells and double-negative 2 B cells, have never been examined in lupus synovial tissue, so that every cellular account of this compartment remains an extrapolation from rheumatoid arthritis. Recent large-cohort data nevertheless indicate that lupus arthropathy is not a single entity, since non-deforming arthritis and Jaccoud’s arthropathy are both associated with a high type I interferon signature whereas arthritis with clinically irreducible deformity is interferon-low, although whether these represent distinct biological trajectories or successive stages of a single process remains to be established. This heterogeneity is invisible to the instruments used in practice, because the composite indices that grade disease activity and determine eligibility for advanced therapy depend on the detection of joint swelling at the bedside, while musculoskeletal ultrasound and contrast-enhanced magnetic resonance imaging independently demonstrate inflammation in approximately half of patients whose joints appear clinically unremarkable, together with erosive change in patients who lack anti-citrullinated protein antibodies. Misclassification of articular disease is therefore systematic and bidirectional, and the same failure has propagated into drug development, where no recent registrational program has used imaging to define articular response, even though the proportion of patients failing on articular endpoints lies between roughly a third and a half. This review synthesizes the immunopathological, biomarker, imaging and therapeutic literatures, and argues that single-cell interrogation of the lupus synovium, a lupus-specific imaging scoring system and a validated imaging-anchored articular outcome measure are the necessary next steps if this manifestation is to be treated with the precision now applied to lupus nephritis.

## 1 Introduction

Musculoskeletal involvement is among the most common manifestations of systemic lupus erythematosus (SLE), reported in up to 95% of patients over the course of their disease, with approximately half experiencing joint pain as their presenting symptom (1, 2). Its impact is significant, with joint symptoms reported as a dominant determinant of health-related quality of life and functional impairment in the disease (3). In a European survey of 4,375 patients, joint pain and swelling were identified both as among the most difficult symptoms to live with and among the symptoms patients would most want eliminated (4). Arthritis is also embedded in the classification of the disease itself, with the 2019 European Alliance of Associations for Rheumatology (EULAR)/American College of Rheumatology (ACR) criteria defining joint involvement as synovitis in two or more joints characterized by swelling or effusion, or tenderness in two or more joints accompanied by at least 30 min of morning stiffness (5), while the SLE Disease Activity Index 2000 (SLEDAI-2K) requires more than two joints with pain and objective signs of inflammation to be characterized as active within the arthritis domain (6).

The long-standing characterization of lupus arthropathy as a benign entity has been questioned for more than a decade (7), and the imaging evidence (reviewed in this manuscript) has largely settled the question. The paradox is that one of the most prevalent manifestations of SLE is also one of the least well instrumented. Lupus nephritis has a tissue-based classification, a histological activity and chronicity index, validated surrogate endpoints, and a regulatory pathway built around them. Lupus arthritis has none of these. There is no organ-specific articular index, no validated imaging scoring system, and no agreed definition of what articular remission means. Assessment rests instead on the musculoskeletal domains of composite instruments that were designed to capture systemic disease and that depend, ultimately, on whether a clinician can detect a swollen joint.

Several reviews have addressed lupus arthritis in recent years, covering its clinical assessment (8), its place among the musculoskeletal manifestations of the disease (9), and the imaging literature specifically (10, 11). The present appraisal is not intended to duplicate them; instead, its purpose is to examine why a manifestation affecting the great majority of patients continues to be assessed with instruments designed to measure something else, to establish how much of the immunological account of the lupus joint rests on direct observation of lupus joint tissue rather than on inference from rheumatoid arthritis, and to follow the consequences of that measurement failure from the clinic into late-phase drug development.

## 2 The clinical spectrum of lupus arthropathy

Terminology in this field is frequently applied inconsistently, and the following convention is used throughout. Lupus arthropathy denotes the whole spectrum of articular involvement attributable to SLE, whether or not deformity is present. Lupus arthritis denotes inflammatory joint disease within that spectrum, whether clinically apparent or detectable only on imaging. Deforming arthropathy denotes any lupus-associated joint deformity, of which Jaccoud’s arthropathy, defined by reducibility and by the absence of erosion on plain radiography, is one form, and clinically irreducible deformity, which encompasses rhupus, is another.

Joint involvement in SLE most often presents as a symmetrical, transient and migratory polyarthralgia. Persistent inflammatory arthritis occurs in a minority, and tenosynovitis is a prominent and under-recognized component, reported in between 10% and 44% of patients (12). The traditional teaching that lupus arthritis is non-deforming and non-erosive is now understood to describe only the most common of several phenotypes. Van Vugt and colleagues distinguished three patterns of deforming arthropathy, comprising mild non-erosive deformity, Jaccoud’s arthropathy and erosive disease of rhupus type (13).

Jaccoud’s arthropathy, defined by reducible deformities—including ulnar drift, swan-neck and boutonnière deformities, z-shaped thumb, and limited metacarpophalangeal extension—that occur in the absence of erosions on plain radiographs, and affects up to 15% of patients (2). Its mechanism is understood to be periarticular and capsular laxity resulting from recurrent tenosynovitis rather than from synovial invasion of bone. The Jaccoud’s arthropathy index of Spronk and colleagues, which scores these five features and sets a threshold of more than five points for definite disease, remains the only dedicated instrument (14). It has never been validated in a large cohort and assesses the hands alone. Importantly, the “non-erosive” designation is a statement about plain radiography rather than about pathology, since higher-resolution modalities (such as MRI and ultrasound) detect small erosions in a proportion of these patients.

Rhupus, in which chronic synovitis produces erosive change indistinguishable from rheumatoid arthritis (RA), affects fewer than 5% of patients with SLE (2). It is more frequent in women, and in most series the articular syndrome precedes the diagnosis of SLE by several years (15, 16). This classification has recently been placed on a much firmer empirical footing. In a cohort of 2,264 patients from the Toronto Lupus Clinic, arthritis was documented in 1,248 (55.1%), of whom 908 (72.6%) had non-deforming arthritis, 239 (19.2%) Jaccoud’s arthropathy, and 101 (8.1%) arthritis with clinically irreducible deformities (17). The immunological findings from that study are more interesting still and are considered below. The three phenotypes, their relative prevalence within that cohort, and their imaging and serological correlates are summarized in **Figure 1**.

**Figure 1:**
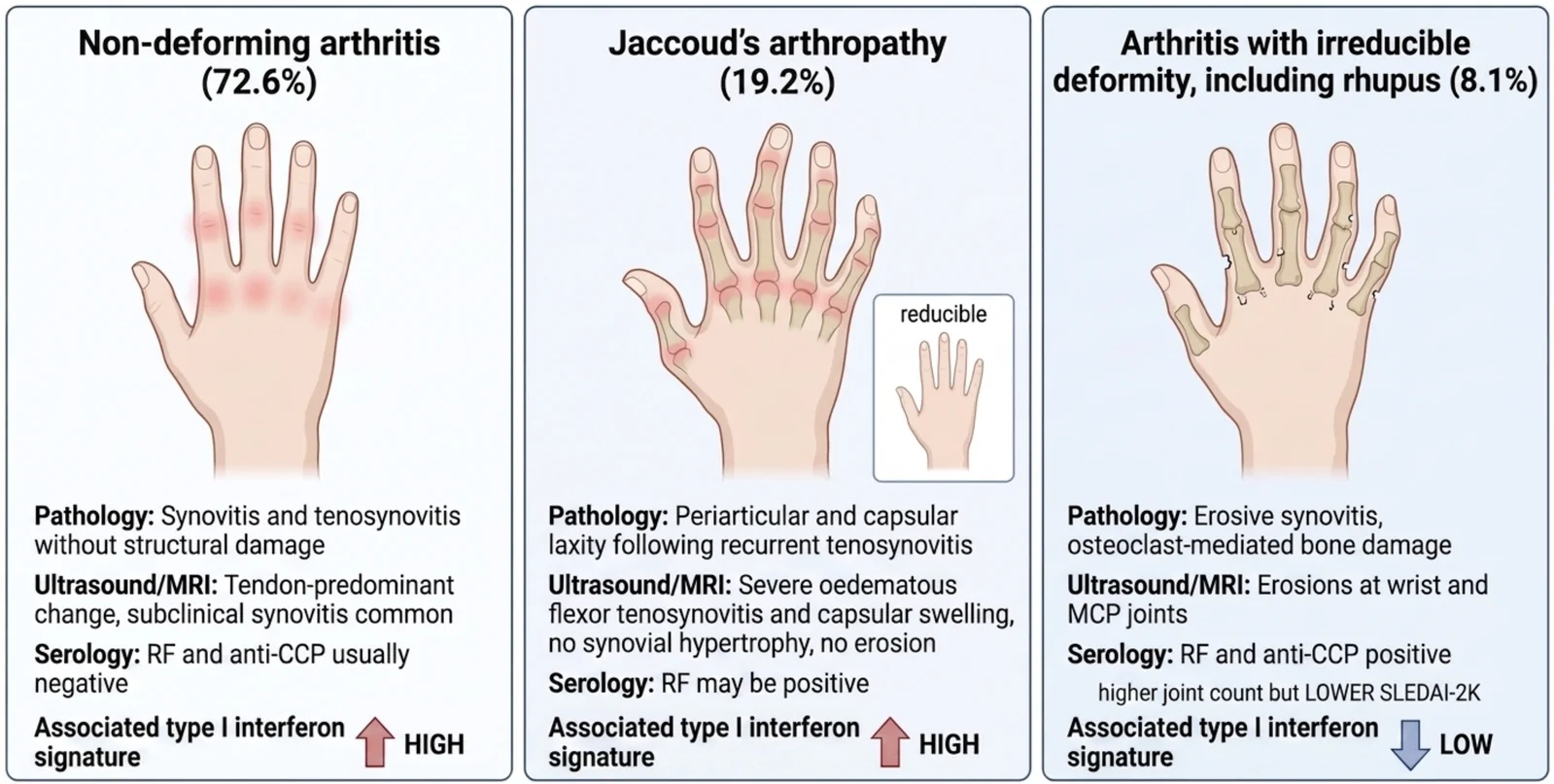
The spectrum of lupus arthropathy. Three phenotypes are distinguished by deformity, reducibility and erosion. Non-deforming arthritis (72.6% of patients with lupus arthritis) is associated with a high type I interferon signature and tendon-predominant ultrasound change. Jaccoud’s arthropathy (19.2%) produces reducible deformity through periarticular and capsular laxity following recurrent tenosynovitis, with severe edematous flexor tenosynovitis and capsular swelling on MRI in the absence of synovial hypertrophy, and is also interferon-high. Arthritis with clinically irreducible deformities, encompassing rhupus (8.1%), has been reported to show erosive synovitis, rheumatoid factor and anti-CCP positivity, and, in contrast to the other two phenotypes, a low interferon signature and lower SLEDAI-2K despite a higher joint count. Interferon status is presented as an association observed at the point of assessment rather than as a demonstrated causal driver of phenotype, and the cross-sectional design of the source cohort cannot establish whether the low signature accompanying irreducible deformity reflects a separate mechanism or a later stage of a single process. Prevalence figures derive from a cohort of 2,264 patients.

A confounder that pervades every discussion of lupus arthritis deserves explicit statement at the outset. Fibromyalgia is present in a pooled 15.8% (95% CI 13.4–18.5) of patients with SLE, with an odds ratio of 3.7 (95% CI 2.74–5.0) relative to controls, though with very high between-study heterogeneity (18). Concomitant fibromyalgia inflates patient-reported and physician-assessed measures of lupus activity independently of inflammatory burden (19). Any instrument that relies on tenderness rather than on objective inflammation will therefore misattribute a substantial fraction of articular symptom burden.

## 3 Immunopathology of the lupus joint

The immunopathology of lupus arthritis is conventionally defined by contrast with RA, as a process driven by immune complexes and type I interferon (IFN) rather than by the anti-citrullinated protein antibody (ACPA)-dependent pathway that characterizes RA. Direct evidence obtained from lupus synovial tissue is limited, and the account that follows distinguishes throughout between mechanisms demonstrated in the lupus joint, those inferred from other manifestations of SLE, and those extrapolated from RA. **Table 1** grades the principal mechanistic claims on that basis, and **Figure 2** applies the same distinction to the cellular composition of the two synovia.

**Table 1:** Strength of the evidence underlying the principal mechanistic claims about the lupus joint.

Claim about the lupus joint	Level of evidence	Source of the evidence	Principal limitation
Type I interferon-inducible transcripts are present in lupus synovial tissue	Directly demonstrated in lupus joint tissue	Microarray of needle-arthroscopic knee biopsies from six patients with untreated SLE, confirmed by RT-PCR, and tubuloreticular structures in seven of 17 synovial fluids	Single small cohort, knee tissue only, no confirmation by single-cell or spatial methods
The lupus synovium is myeloid-dominated rather than lymphocyte-dominated	Directly demonstrated, though by deconvolution rather than by cell counting	Computational reanalysis of legacy bulk transcriptomic datasets	Deconvolution estimates proportions and cannot exclude a quiescent lymphocyte population, and digestion of small samples recovers lymphocytes less efficiently
Ectopic germinal centers are absent from the lupus synovium	Directly demonstrated	Absence of *in situ* germinal center signature on transcriptomic reanalysis, consistent with scant infiltrate on histology	Absence of a transcriptional signature is weaker evidence than direct histological search in an adequately powered series
Fibroblast activation protein alpha expression is reduced relative to comparators	Directly demonstrated	Microarray comparison with RA and osteoarthritis	Single dataset, no protein-level or single-cell confirmation
IL-6 and IL-17A are produced within the articular compartment	Directly demonstrated	Synovial fluid concentrations exceeding paired plasma in two independent small series	Fluid rather than tissue, small numbers, no cellular source identified
Immune complex deposition and complement consumption contribute to lupus synovitis	Indirect	Association of low C3 and C4 with ultrasound abnormality, and classical descriptions of antinuclear antibodies within joint effusions	Association only, no direct demonstration of complex deposition in synovial tissue by contemporary methods
Interferon status separates arthritis phenotypes	Indirect and cross-sectional	Peripheral blood interferon signature in 475 patients within a cohort of 2,264	Blood rather than joint, single cohort, cross-sectional, cannot distinguish a distinct entity from a late stage of one process
Anti-carbamylated protein antibodies mark erosive articular damage	Indirect	Serological association with ultrasound erosion in two cohorts, with a third reporting lower prevalence	Serum association, no mechanistic demonstration in joint tissue, assays not standardized
T peripheral helper cells participate in lupus joint pathology	Extrapolated	Established in seropositive RA synovium and expanded in SLE blood	Never examined in lupus synovial tissue, and therefore a hypothesis rather than a finding
Double-negative 2 B cells act as synovial plasma cell precursors in SLE	Extrapolated	Established as the principal synovial plasma cell precursor in RA and as a major extrafollicular population in SLE blood	Never examined in lupus synovial tissue, and therefore a hypothesis rather than a finding
Pathogenic fibroblast subsets partition inflammation and damage in the lupus joint	Extrapolated, and the available lupus data point the other way	FAPa and THY1 subsets defined in RA	No equivalent analysis in SLE, and reduced FAPa expression argues against a direct parallel
Plasmacytoid dendritic cells, NETosis and Fc gamma receptor signaling operate within the lupus joint	Untested	Established in other SLE target organs and in the circulation	No synovial data of any kind, and included here to define the research agenda rather than to describe the disease

Claims are graded as directly demonstrated in lupus synovial tissue or fluid, indirect, extrapolated from rheumatoid arthritis, or untested.

**Figure 2:**
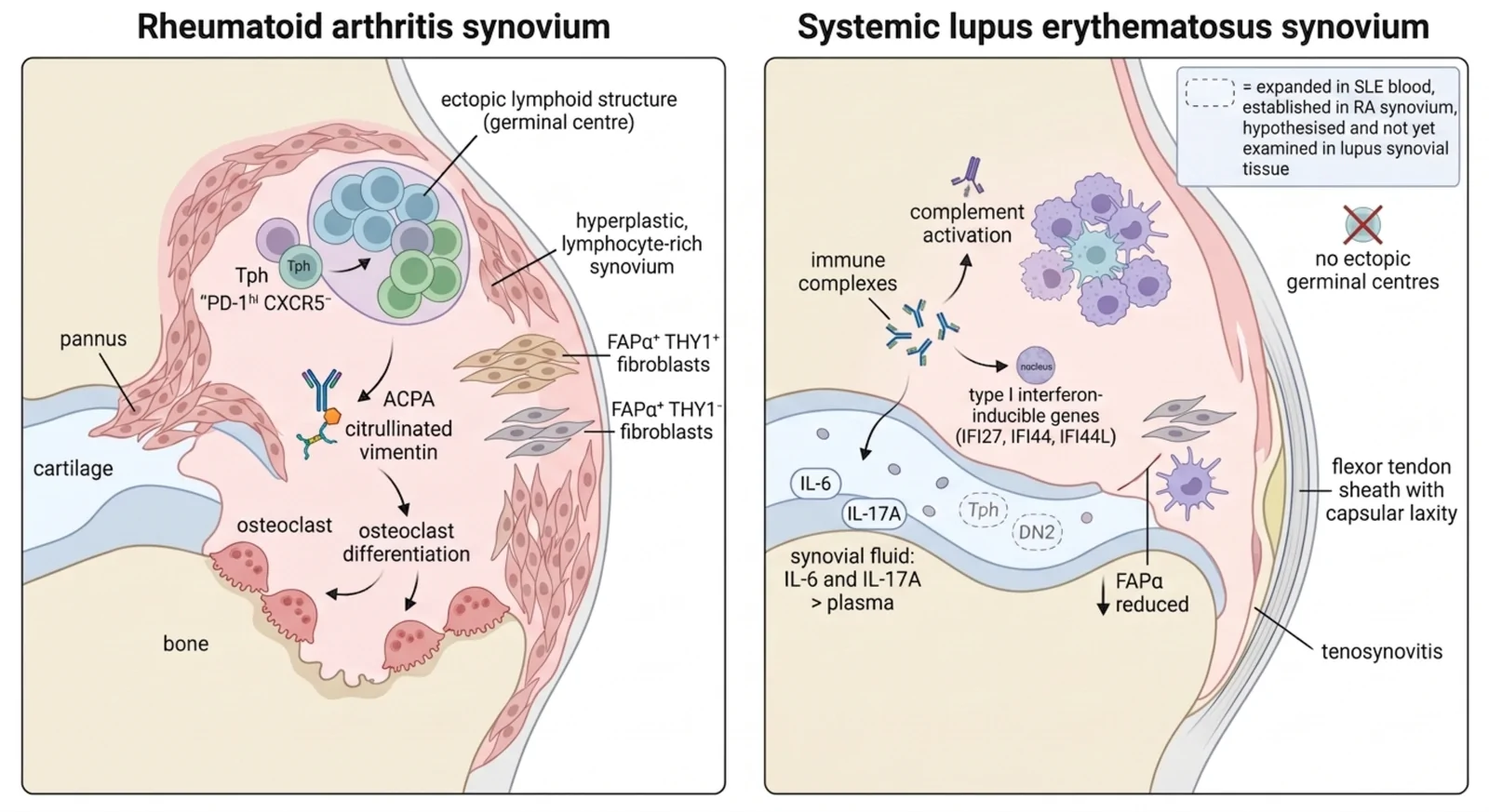
Immunopathology of the lupus joint, contrasted with rheumatoid arthritis. In rheumatoid arthritis (left), anti-citrullinated protein antibodies (ACPA) directly induce osteoclastogenesis, producing erosive bone damage, within a lymphocyte-rich synovium containing ectopic lymphoid structures and an expanded FAPα^+^ pathogenic fibroblast compartment. In systemic lupus erythematosus (right), the synovium is relatively pauci-immune and myeloid-dominated, with type I interferon-inducible gene expression detectable *in situ*, immune complex deposition, and local production of IL-6 and IL-17A within synovial fluid; ectopic germinal centers are not observed, and fibroblast activation protein alpha expression is reduced. Populations shown with dashed outlines (T peripheral helper cells and double-negative 2 B cells) are established in the rheumatoid synovium and expanded in the circulation in SLE but have not been examined in lupus synovial tissue. The dashed outline is used throughout to denote populations that have never been examined in lupus synovial tissue and whose presence in this compartment is therefore hypothetical. Features shown without a dashed outline are those for which direct evidence from lupus joint tissue or synovial fluid exists, graded in **Table 1**.

### 3.1 What the synovial tissue demonstrates in SLE

The entire primary literature on SLE synovial tissue rests on limited datasets. Nzeusseu Toukap and colleagues performed microarray analysis of needle-arthroscopic knee synovial biopsies from six patients with untreated SLE, compared with seven with RA and six with osteoarthritis. They demonstrated upregulation of IFN-inducible genes (*IFI27*, *IFI44*, and *IFI44L*) together with downregulation of genes governing extracellular matrix homeostasis (20). A subsequent computational reanalysis by Hubbard and colleagues, applying differential expression, weighted gene co-expression network analysis, and gene set variation analysis to the same and comparable legacy datasets, concluded that lupus arthritis is dominated by a myeloid and macrophage transcriptional phenotype, in contrast to the lymphocyte-dominated pattern of RA and the fibroblast-dominated pattern of osteoarthritis. They also found no evidence of *in situ* germinal center formation within the joint (21). The only substantial histological series remains a 1991 study of 30 percutaneous synovial biopsies, which described synoviocyte hyperplasia, vascular proliferation, and fibrinoid change with a conspicuously scant inflammatory infiltrate, and in which direct immunofluorescence was not discriminatory (22).

These datasets support the recurring characterization of the lupus synovium as relatively pauci-immune, myeloid-skewed, and interferon-imprinted, although they derive from a small number of patients studied by methods that predate the current era, and no single-cell RNA sequencing or mass cytometry study of SLE synovial tissue has yet been reported. The Accelerating Medicines Partnership programs that transformed understanding of the rheumatoid synovium enrolled only rheumatoid and osteoarthritic tissue (23, 24), and the SLE arm of that program profiled kidney rather than joint. Every cellular hypothesis about the lupus joint discussed below is therefore an extrapolation awaiting its confirmatory experiment.

Two technical caveats attach to the pauci-immune description in particular. Bulk transcriptomic deconvolution estimates the relative contribution of cell populations rather than counting cells, so a myeloid-skewed signature is compatible with a lymphocyte population that is present but transcriptionally quiescent. Enzymatic digestion of small synovial samples also recovers lymphocytes less efficiently than myeloid cells, and the historical biopsies were obtained from knees with effusions large enough to permit needle arthroscopy, which is not the presentation of most lupus arthritis. Low lymphocyte abundance in the lupus synovium may therefore reflect true tissue biology, systematic underdetection, or a combination of the two, and the datasets available cannot distinguish between these possibilities. The description of this compartment as pauci-immune and myeloid-dominated should accordingly be read as a reproducible observation across the datasets that exist rather than as an established property of the tissue.

### 3.2 Mechanisms extrapolated from rheumatoid arthritis

In RA, ACPA are not merely diagnostic but directly osteoclastogenic. Antibodies against citrullinated vimentin induce osteoclast differentiation and bone loss *in vitro* and *in vivo*, providing a mechanistic explanation for the erosive phenotype (25–27). The general absence of ACPA in SLE is the most parsimonious explanation for why erosive change is comparatively uncommon in lupus arthritis, and, conversely, why ACPA-positive patients with SLE tend towards a rhupus phenotype.

Two cellular populations of considerable interest in SLE have an established role in the RA joint but have not been examined in the lupus synovium. T peripheral helper (Tph) cells (PD-1^hi^ CXCR5^−^CD4^+^ T cells that provide B cell help outside follicular structures) were first described as a pathologically expanded population in seropositive RA synovium (28). Tph cells are expanded in the circulation in SLE, produce interleukin (IL)-21, and correlate both with disease activity and with the frequency of CD11c^+^ B cells (29, 30). Similarly, the Toll-like receptor 7 (TLR7)-driven double-negative 2 (DN2) B cell subset that constitutes a major extrafollicular effector population in SLE (31) has been identified as the principal synovial plasma cell precursor in RA (32). For both populations, the evidence is divided between diseases, with the lupus data linking them to global disease activity and to nephritis, and the synovial data deriving exclusively from RA. Neither population has been examined in lupus synovial tissue, and their inclusion in any account of the lupus joint is at present a hypothesis rather than a confirmed finding. The extrafollicular character of the lupus B cell response, together with the absence of germinal center organization in the lupus synovium, makes this compartment a direct test of whether these populations operate in the joint.

The stromal compartment shows a similar asymmetry, as in RA, fibroblast subsets defined by fibroblast activation protein alpha (FAPα) and THY1 partition the pathology, with FAPα^+^THY1^+^ cells driving inflammation and FAPα^+^THY1^−^ cells driving bone and cartilage damage (33, 34). No equivalent analysis has been conducted to date in SLE. What data there are point in the opposite direction, with FAPα expression noted to be significantly downregulated in the SLE synovium relative to comparators (20), and lupus arthritis segregated as myeloid- rather than fibroblast-dominated (21). The available evidence therefore suggests that the expanded pathogenic fibroblast compartment characteristic of RA is not a feature of the lupus joint, although more detailed analysis is required in order to fully test this finding across lupus arthropathies more generally.

### 3.3 Cytokines and the role of interferon

IL-6 is the cytokine with the most consistent articular signal in SLE, and its biology in synovitis is well established. It drives synovial angiogenesis through modulation of angiopoietin expression (35), and serum concentrations behave as a surrogate for synovial inflammation in early RA (36). IL-17A has been detected at elevated concentration in SLE synovial fluid (37), but the systemic data are equivocal and, where clinical associations exist, they point elsewhere. In 98 patients studied cross-sectionally and 75 longitudinally, serum IL-17 was elevated in SLE relative to healthy donors but did not correlate with disease activity as measured by SLEDAI-2K and was specifically associated with neuropsychiatric rather than articular disease (38). IL-17-producing double-negative T cells in SLE have been localized to the kidney (39). A joint-specific IL-17 axis in SLE is therefore plausible based on synovial fluid data but remains unproven and should not be asserted.

The type I interferon (IFN-I) story has become considerably more nuanced over recent years. A peripheral blood IFN-I signature is present in half to three-quarters of patients with SLE, with the range reflecting assay, cohort and ancestry. The original description reported the signature in about half of patients with severe disease (40), a contemporary cohort of 506 patients found 57.5% (41), and the 823-patient SPOCS cohort found 70.6%, rising to 83.3% in Asian and 86.5% in Black patients (42). IFN-I is detectable in SLE synovial tissue (20) and promotes antigen presentation, B cell survival, and myeloid activation. It does not, however, follow that IFN-I drives lupus arthritis. Neither SPOCS nor the Toronto cohort identified musculoskeletal involvement among the domains distinguishing interferon-high from interferon-low patients (41, 42), and systemic IFN activity in treatment-naive patients tracks with global activity and damage accrual rather than with articular disease specifically (43).

The most informative data come from the arthritis-subtype analysis of 2,264 patients referred to above. Within the 475 patients with interferon data, non-deforming arthritis and Jaccoud’s arthropathy were both associated with a high IFN-I signature, whereas arthritis with clinically irreducible deformities was associated with a low signature (17). The deforming phenotype also carried a higher adjusted joint count alongside a lower adjusted SLEDAI-2K, together with associations to neurological involvement and anti-Ro antibodies. Taken together, these findings are consistent with lupus arthropathy comprising at least two potentially distinct biological trajectories rather than a single disease with a spectrum of severity: an IFN-associated inflammatory synovitis encompassing both non-deforming disease and Jaccoud’s arthropathy, alongside a lower-inflammation deforming process whose pathogenesis remains unexplained. That interpretation requires validation and is not the only one available. Because the data are cross-sectional, a low IFN signature in patients with fixed deformity is equally compatible with a late stage of the same process, in which transcriptional inflammatory activity has declined through remodeling, fibrosis, cumulative treatment exposure, or disease burnout after the structural damage has been established. Distinguishing a separate pathogenic entity from a late stage of a shared process requires longitudinal IFN measurement across the evolution of deformity, together with tissue from patients at each stage, and neither is currently available. What can be said is that the two groups differ at the point of assessment in a way that would be expected to affect their response to IFN-directed therapy, which is a sufficient reason to stratify for arthritis phenotype in trials of such agents.

### 3.4 Mechanisms that remain untested in the lupus joint

The account above is dominated by type I IFN, IL-6, IL-17, and immune complexes because those are the mediators for which any lupus joint data exist. Several pathways of established importance elsewhere in SLE have never been examined in synovial tissue, and they are set out here as testable propositions rather than as mechanisms of lupus arthritis. Plasmacytoid dendritic cells are the principal source of type I IFN in SLE and are activated through TLR7 and TLR9 by nucleic acid-containing immune complexes, with TLR7 gene dosage raised in women through escape from X-chromosome inactivation (44). Whether plasmacytoid dendritic cells are recruited to the lupus synovium, and whether the IFN-inducible transcripts detected in that tissue are produced locally or reflect a systemic signature imprinted on resident cells, has not been determined. Neutrophil extracellular traps (NETs) enriched in oxidized mitochondrial DNA are interferogenic and contribute to lupus-like disease in animal models (45), and neutrophils are abundant in inflamed synovial fluid, yet NETosis has not been quantified in the lupus joint.

Immune complex deposition is the mechanism most often invoked to explain lupus synovitis, and the observation that low C3 and C4 concentrations are associated with a higher probability of ultrasound abnormality is consistent with local complement consumption (46). The downstream biology, comprising Fc gamma receptor engagement, the balance between activating and inhibitory receptors, and the efficiency with which complexes and apoptotic material are cleared, has been characterized in the kidney and the circulation but not in the joint. The clinical relevance of this axis is no longer hypothetical, since obexelimab, a bifunctional antibody that co-engages CD19 and the inhibitory receptor Fc gamma receptor IIb, has been evaluated in SLE (47).

Tissue-level organization is the largest gap of all. Synovial macrophages in rheumatoid arthritis partition into subsets whose relative abundance distinguishes active disease from sustained remission (48), and integrated single-cell and spatial analysis has resolved the rheumatoid synovium into reproducible cellular neighborhoods that predict treatment response (24). The equivalent analysis in lupus has been performed in the kidney, where the Accelerating Medicines Partnership defined an immune cell landscape with prognostic value (49), but not in the joint. Whether the lupus synovium contains organized immune niches, how stromal and immune populations interact within it, and whether tissue-resident populations persist there between flares are all open questions, and each is answerable with methods that are already in routine use for other tissues.

## 4 Biomarkers of articular disease

### 4.1 Serum biomarkers

Autoantibody associations with deforming arthropathy are established but narrower than often stated. In a study of 34 patients with SLE, rheumatoid factor was present in 65% of those with deforming arthropathy compared with 15% of those without (p = 0.005), whereas anti-cyclic citrullinated peptide (anti-CCP) antibody positivity did not differ between the deforming and non-deforming groups, at 7% and 5%, respectively (50). A larger Chinese cohort of 267 patients reached an apparently divergent conclusion, finding anti-CCP antibodies more prevalent in patients with arthritis than without, and markedly higher titers in the 12 patients with erosive arthropathy than in those without erosions (221 ± 88 vs. 32 ± 42 U/mL, p <0.05) (51). The tension between these studies is instructive rather than contradictory. Rheumatoid factor appears to mark deformity in general, whereas anti-CCP marks the specifically erosive, rhupus-like subset, which in any individual cohort is small. The erosive-group estimate rests on 12 patients and should be treated accordingly.

Antibodies against carbamylated proteins deserve particular attention, since they offer a candidate explanation for erosive change in patients who are ACPA-negative. In 78 patients with SLE and joint involvement, anti-carbamylated protein antibodies were present at a prevalence comparable to that seen in RA and significantly higher than in patients with SLE without joint involvement or in healthy controls, and 45% of patients negative for both ACPA and rheumatoid factor were positive for these antibodies (52). In a larger cohort of 152 patients with articular involvement, prevalence was 28.3%, comparable to rheumatoid factor at 27.6% and significantly higher than ACPA at 11.2%, and positivity was associated with ultrasound-detected erosive disease, with a correlation between antibody titer and erosive score (52). A study of two well-characterized European cohorts reported a lower prevalence, indicating that the size of the association remains uncertain (53), and a machine-learning analysis identified anti-carbamylated protein antibodies among the variables contributing to prediction of the erosive phenotype (52). Taken together, these data suggest that carbamylation, rather than citrullination, may be the post-translational modification relevant to bone damage in lupus arthritis, which would reconcile the finding of erosions in ACPA-negative patients discussed below with the general absence of an ACPA-driven mechanism in this disease.

Among soluble mediators, IL-6 has the most reproducible articular association. In a Norwegian cohort of 47 patients, IL-6 concentrations were significantly higher in those with active or deforming joint disease than in those with prior or no arthritis (54). Serum IL-6 also correlated strongly with total ultrasound activity score, whereas tumor necrosis factor (TNF) and B cell activating factor did not (55). The frequently repeated claim that IL-6 correlates with power Doppler signal requires qualification. The supporting evidence comes from juvenile chronic arthritis (56), and a study of normal joints found that grayscale scores correlated with no pro-inflammatory cytokine and that power Doppler correlated with vascular endothelial growth factor rather than IL-6 (57).

C-X-C motif chemokine ligand 13 (CXCL13) has been proposed as a marker of tenosynovitis, but the primary study is a negative one at the level of arthritis, with no association found between CXCL13 concentration and the SLEDAI arthritis descriptor, clinically swollen joints, or ultrasound-detected synovitis. The authors concluded that CXCL13 does not appear to be a biomarker for arthritis in this population (58). Any residual association with tenosynovitis specifically requires independent replication before it can be regarded as established.

### 4.2 Synovial biomarkers

Synovial fluid analysis in SLE has been limited by the practical difficulty that large effusions are an uncommon presentation of joint involvement in the disease. The classical description emphasizes lymphocyte predominance with lupus erythematosus cells (59) and the detection of antinuclear antibodies within joint effusions (60). Leukocyte counts in SLE synovial fluid range from 875 to 39,250 cells/mm³, a spread that the lymphocyte-predominant classical description does not convey. The same study reported tubuloreticular structures, a morphological correlate of local IFN-I exposure, in seven of 17 samples, an observation that anticipates the transcriptomic interferon signature by more than a decade and that remains unexamined by modern methods (59).

Cytokine analyses consistently show compartmentalization, with synovial fluid IL-6 concentrations exceeding plasma concentrations, whereas synovial TNF and B cell activating factor are equivocal or lower (55). A study of synovial fluid from 17 patients identified higher IL-6 and IL-17A in fluid than in plasma (37). The consistent finding across small studies is that the articular compartment is not simply a passive reflection of systemic disease, which is precisely why systemic disease activity indices should not be expected to capture it, and why serum biomarkers alone are unlikely to suffice. Studies reporting different serum and synovial biomarkers in SLE patients with MSK involvement are summarised in **Table 2**.

**Table 2:** Serum and synovial biomarkers studied in relation to musculoskeletal disease in systemic lupus erythematosus.

Author (year)	Country	Patients	Compartment	Biomarker	Key finding
Schumacher and Howe (1995) (59)	USA	17 SLE synovial fluids	Synovial	Cellular morphology	Leukocyte counts spanned a wide range (875–39,250 cells/mm³). LE cells in 8/17 fluids; tubuloreticular structures, a morphological correlate of local type I interferon exposure, in 7/17.
Damián-Abrego et al. (2008) (50)	Mexico	34 SLE	Serum	Rheumatoid factor, anti-CCP	RF positive in 65% of patients with deforming arthropathy versus 15% without (p = 0.005). Anti-CCP concentrations were normal in both groups (positivity 7% vs. 5%).
Qing et al. (2009) (51)	China	267 SLE	Serum	Anti-CCP	Anti-CCP more prevalent in patients with arthritis than without. Titers markedly higher in the 12 patients with erosive arthropathy than in those without erosions (221 ± 88 vs. 32 ± 42 U/mL, p <0.05).
Eilertsen et al. (2011) (54)	Norway	47 SLE	Serum	IL-6	IL-6 significantly higher in patients with ongoing or deforming joint disease than in those with prior arthritis or none.
Ball et al. (2014) (55)	United Kingdom	50 SLE, 40 RA (synovial subset n = 3)	Serum and synovial	IL-6, TNF-α, BAFF	Plasma IL-6 correlated with total ultrasound activity score; TNF-α and BAFF did not. Synovial fluid IL-6 exceeded plasma concentrations, whereas synovial TNF-α was lower or equivocal.
Ribeiro et al. (2018) (58)	Brazil	64 SLE	Serum	CXCL13, anti-dsDNA, anti-Sm	No association between CXCL13 and the SLEDAI arthritis descriptor, clinically swollen joints or ultrasound synovitis. The authors concluded that CXCL13 does not appear to be a biomarker for arthritis in this population.
Sippl et al. (2021) (37)	Sweden	17 SLE	Synovial	IL-6, IL-17A	IL-6 and IL-17A concentrations higher in synovial fluid than in plasma, indicating local production within the articular compartment.

## 5 Musculoskeletal imaging in SLE

### 5.1 Ultrasound

Musculoskeletal ultrasound is safe, non-invasive, repeatable, and inexpensive relative to MRI, and power Doppler adds sensitivity for the soft-tissue hyperemia that accompanies active inflammation. It should be noted that almost the entire SLE literature has been generated using conventional grayscale and power Doppler equipment rather than the high-frequency or high-resolution transducers that the term high-resolution ultrasound properly denotes, and the findings summarized below should be read accordingly (61). It depicts the periarticular and intra-articular pathology of SLE, including the presence of synovitis, tenosynovitis, erosion and tendon rupture (62), although the definitions applied are those developed by OMERACT for RA rather than for SLE. Those definitions were derived and validated against the dominant lesion of rheumatoid disease, which is proliferative synovial hypertrophy with Doppler signal, whereas the dominant lesion in lupus is tenosynovial and capsular. An instrument calibrated against one lesion will not necessarily quantify the other, an important limitation given the biological differences in synovial pathology described above (63). A systematic review of the field concluded that ultrasound consistently detects abnormality beyond that identified clinically, but that heterogeneity of scanning protocols and scoring precludes pooled estimates (64), and the position has not materially changed since.

A systematic review and meta-analysis of advanced imaging in lupus arthritis, restricted for ultrasound to studies published between 2019 and 2024, included 16 ultrasound studies, eight MRI studies, and a single optical imaging study (10). Pooled analysis showed a strong association between joint symptoms and ultrasound synovitis, with an odds ratio of 10.89 (95% CI 4.02 to 29.48), and between joint symptoms and tenosynovitis, with an odds ratio of 5.93 (95% CI 1.99 to 17.72). The association weakened to 5.00 (95% CI 1.11 to 22.60) when the analysis was restricted to physician-confirmed arthritis, which is consistent with clinical assessment adding noise rather than precision. The authors concluded that the evidence does not yet support the use of advanced imaging as an outcome measure, a conclusion that this review shares and that defines the work that remains. A more recent scoping review has separately catalogued the burden of enthesitis, inflammatory arthritis, and tenosynovitis detectable by ultrasound in this disease (65), and a narrative synthesis has summarized recent methodological advances in both modalities (11).

The wrists, metacarpophalangeal and proximal interphalangeal joints are the most frequently abnormal sites (46, 62, 66), though metatarsophalangeal involvement may exceed that of the hands and wrists when systematically sought (67). Effusion frequency in the knee is comparable between SLE and RA, whereas synovial proliferation and power Doppler positivity are more prevalent in RA, a pattern consistent with either a milder synovitis in SLE or one differing in cellular composition (68). Ultrasound also discriminates between the clinical phenotypes, with erosive change concentrating in rhupus and tendon abnormality predominating in Jaccoud’s arthropathy and in non-deforming, radiographically non-erosive disease (46, 58). Power Doppler positivity is more frequent in patients with clinically active disease, whereas subclinical or inactive disease has been reported to show tendon-predominant change (46).

Ultrasound has also identified articular pathology that the conventional description of lupus arthritis does not include. Among 106 patients with SLE undergoing articular ultrasound for tender or swollen joints, enthesitis was present in 31 (29.2%), and over a median follow-up exceeding 10 years, those patients developed renal involvement less frequently than controls without enthesitis and responded poorly to B cell depletion (69). The observation requires replication, and the retrospective design and the referral of patients for scanning on clinical grounds both limit inference. It nonetheless illustrates that imaging may resolve phenotypes with distinct trajectories and distinct treatment responses, which is the practical argument for an imaging-anchored articular assessment rather than a merely descriptive one.

The most consequential ultrasound finding, however, concerns the patients in whom clinical examination is unremarkable. Subclinical synovitis is present in 58.3% of asymptomatic patients with SLE. Ultrasound synovitis score correlates modestly with erythrocyte sedimentation rate (r = 0.30, p <0.05) and anti-double-stranded DNA titer (r = 0.34, p <0.05). Furthermore, 22.9% of those with subclinical synovitis developed new musculoskeletal symptoms within 6 months (70). Ultrasound abnormality is also common in patients whose joints are neither tender nor swollen on examination (71).

This discordance was quantified definitively by Zayat and colleagues, who studied 88 consecutive patients with SLE and inflammatory musculoskeletal symptoms alongside 24 asymptomatic controls, having deliberately excluded patients positive for rheumatoid factor or anti-CCP so that an occult rhupus subgroup could not explain the findings (72). Ultrasound-confirmed inflammation, including grayscale ≥2 with power Doppler ≥1, or tenosynovitis was present in 68% of symptomatic patients. Yet only 38% met British Isles Lupus Assessment Group (BILAG) A or B criteria and only 32% met the SLEDAI musculoskeletal criterion. Twenty-seven percent had subclinical synovitis, defined as ultrasound abnormality in the absence of any clinically swollen joint, which was associated with higher serum IgG and higher tender joint counts. Swelling-based classification therefore misses approximately half of patients with objectively inflamed joints while simultaneously capturing patients whose symptoms have no objective inflammatory correlate, so that its use to select patients for clinical trials or for treat-to-target escalation undertreats the first group and exposes the second to immunosuppression from which no articular benefit can follow.

The therapeutic implication of this discordance has since been tested prospectively. In the Ultrasound Evaluation for Musculoskeletal Lupus study, 133 patients with physician-diagnosed inflammatory joint pain received a single intramuscular dose of methylprednisolone and were assessed clinically and by ultrasound at baseline, 2 weeks, and 6 weeks (73). Seventy-eight had a positive scan, of whom only 53 (68%) had any swollen joint, while 20% of the 66 patients with a swollen joint had a negative scan, which reproduces the bidirectional misclassification described above in a prospective design. The primary endpoint was not met in the full analysis set, but after exclusion of the 32 patients with fibromyalgia, those with a positive baseline scan showed significantly greater improvement in early morning stiffness at two weeks, and the difference increased when adjusted for treatment received. BILAG and SLEDAI responses were also higher in ultrasound-positive patients. Two conclusions follow from this trial that could not be drawn from cross-sectional imaging studies. Imaging-defined synovitis predicts response to treatment in a way that clinical examination does not, and the presence of fibromyalgia is sufficient to conceal that relationship in an unselected population, which is precisely the dilution effect that afflicts trial cohorts assembled on the basis of tenderness and swelling.

Consistent with this, several studies have found no association between ultrasound findings and SLEDAI-2K or BILAG scores (67, 68). Notably, however, RA-derived composite indices perform better than SLE-specific ones. In 106 patients, ultrasound score correlated with DAS28-CRP (r = 0.3), the simplified tender-swollen ratio (r = 0.42), Simplified Disease Activity Index (SDAI) (r = 0.33), and Clinical Disease Activity Index (CDAI) (r = 0.29) (74). That an instrument designed for a different disease outperforms the lupus-specific indices at measuring the lupus joint is, in itself, an argument for developing a dedicated articular measure.

Ultrasound criteria for lupus arthritis therefore remain undefined. What is required is not further demonstration that abnormality exists, but characterization of which findings correspond to clinically meaningful arthritis, tenosynovitis, and erosion in SLE specifically, how these should be quantified, and what their predictive value is for subsequent structural damage.

### 5.2 Magnetic resonance imaging

MRI detects osteitis with high sensitivity, and gadolinium enhancement further improves detection of synovitis, bone marrow edema, tenosynovitis, and peritendonitis (75–77). In Jaccoud’s arthropathy, the characteristic appearance is severe edematous tenosynovitis, predominantly of the flexor tendons, with capsular swelling in the absence of erosion. In the subgroup meeting Jaccoud’s criteria, synovial membrane hypertrophy was absent, supporting a mechanism of pericapsular and tendon fibrosis rather than of proliferative synovitis (78).

Validated MRI measures of inflammation exist for RA in the form of the OMERACT RA MRI Scoring System (RAMRIS), which grades erosion, bone marrow edema, and synovitis, supplemented by a tenosynovitis score (79, 80). The same objection applies to RAMRIS as to the OMERACT ultrasound definitions, in that its components were selected to capture erosion, osteitis, and synovial hypertrophy in a disease where those are the principal lesions. Applied to lupus arthritis, RAMRIS failed to capture the features responsible for patient symptoms, leading investigators to conclude that an SLE-specific MRI scoring system is required (81).

MRI has also revised understanding of erosive disease in SLE. In a study of 34 symptomatic patients, wrist synovitis was present in all and metacarpophalangeal synovitis of at least grade 1 in 93%, with a higher frequency of radiocarpal inflammation than in the RA comparison group (82). Critically, only three of the 34 patients (8.8%) were ACPA-positive, yet erosions were detected at the wrist in 93% and at the metacarpophalangeal joints in 61%. Erosive disease in SLE is therefore neither confined to rhupus nor dependent on ACPA positivity, a finding that plain radiography could not have produced and that materially alters how “non-erosive” arthropathy should be understood in SLE. A negative correlation between anti-RA33 titer and metacarpophalangeal and proximal interphalangeal bone edema was also observed (82).

The largest contrast-enhanced series studied 83 patients with SLE (31 with clinical arthritis, 31 with arthralgia alone, and 21 asymptomatic) alongside 24 healthy controls (77). Synovitis was more frequent in symptomatic patients than in asymptomatic patients or controls, with the radiocarpal-radioulnar joint most commonly affected (45.1%), followed by the intercarpal joint (43.9%). Among patients with arthralgia but no clinical signs of arthritis, 51.6% had MRI synovitis. Tenosynovitis (predominantly of the flexor tendons), peritendonitis, and bone marrow edema were all more frequent than in controls. Approximately half of patients with an unremarkable joint examination therefore have demonstrable inflammation on MRI, a proportion closely comparable to that obtained by ultrasound in separate cohorts using an entirely different physical principle of detection.

The ultrasound and magnetic resonance imaging studies considered in this section are summarized in **Table 3**.

**Table 3:** Ultrasound and magnetic resonance imaging studies of joint disease in systemic lupus erythematosus.

Author (year)	Country	Participants	Modality	Sites assessed	Key finding and interpretation
Ostendorf et al. (2003) (78)	Germany	14 SLE	MRI	Hands	Edematous flexor tenosynovitis and capsular swelling without erosion. Synovial membrane hypertrophy present in 10/14 overall but absent in the four patients meeting Jaccoud’s criteria. Interpretation: the dominant lesion in Jaccoud’s arthropathy lies outside the synovial compartment that RA-derived scoring systems were built to grade.
Iagnocco et al. (2004) (66)	Italy	26 SLE	US	Wrists	Synovitis and tenosynovitis frequently detected at the radiocarpal and intercarpal joints. Interpretation: early demonstration that ultrasound detects articular pathology in SLE, though without a clinical comparator to establish added value over examination.
Wright et al. (2006) (62)	United Kingdom	SLE (pictorial series)	US	Hands	Demonstration of synovitis, tenosynovitis, bone erosion and tendon rupture in SLE hands. Interpretation: pictorial series establishing the range of detectable lesions, descriptive rather than quantitative.
Ossandon et al. (2009) (68)	Italy	SLE versus RA	US	Knees	Effusion frequency comparable between SLE and RA, but synovial proliferation and power Doppler positivity more prevalent in RA. Interpretation: consistent with either a milder synovitis in SLE or one differing in cellular composition, and a caution against transferring RA thresholds directly.
Gabba et al. (2012) (46)	Italy	108 SLE	US	Hands, wrists	Erosive change concentrated in rhupus, while tendon abnormality predominated in Jaccoud’s arthropathy and non-deforming disease. Low C3 and C4 associated with a higher probability of abnormal ultrasound. Interpretation: imaging separates the clinical phenotypes, and the complement association is the only joint-level evidence implicating immune complex handling.
Iagnocco et al. (2014) (67)	Italy	SLE	US	Hands, wrists, feet	Metatarsophalangeal joints more frequently abnormal than radioulnocarpal, MCP or PIP joints. No association with SLEDAI-2K or BILAG. Interpretation: hand-and-wrist protocols may systematically under-detect disease, and composite indices do not track imaging burden.
Yoon et al. (2014) (70)	South Korea	Asymptomatic SLE	US	Hands, wrists	Subclinical synovitis in 58.3%. US synovitis score correlated with ESR (r = 0.30) and anti-dsDNA (r = 0.34). 22.9% developed new musculoskeletal symptoms within 6 months. Interpretation: imaging abnormalities in asymptomatic patients carries prognostic information rather than being an incidental findings.
Ball et al. (2014) (82)	United Kingdom	34 SLE, 15 RA	MRI	Hands, wrists	Wrist synovitis in all patients and MCP synovitis of at least grade 1 in 93%. Only 3/34 (8.8%) were ACPA-positive, yet erosions were present at the wrist in 93% and at MCP joints in 61%. Interpretation: erosive disease in SLE is neither confined to rhupus nor dependent on ACPA, which plain radiography could not have shown.
Dreyer et al. (2015) (71)	Denmark	33 SLE, 11 controls	US	Wrists, MCP	Wrist synovitis in 18% of patients without arthralgia, and any US abnormality in 44% to 46% of wrists without clinical tenderness or swelling. Interpretation: quantifies the false-negative rate of clinical examination in unselected patients.
Zollars et al. (2018) (81)	USA	SLE	MRI (contrast-enhanced)	Hands, wrists	RAMRIS failed to capture the features responsible for patients’ symptoms, leading the investigators to conclude that an SLE-specific MRI scoring system is required. Interpretation: the only formal test of an RA-derived scoring system in lupus arthritis, and it failed.
Ceccarelli et al. (2019) (74)	Italy	106 SLE	US	Hands, wrists	US score correlated with DAS28-CRP (r = 0.30), the ratio of swollen to tender joints (r = 0.42), the Simplified Disease Activity Index (r = 0.33) and the Clinical Disease Activity Index (r = 0.29). Interpretation: RA-derived composite indices track imaging burden better than SLE-specific ones, which is itself an argument for a dedicated articular measure.
Zayat et al. (2019) (72)	United Kingdom	88 symptomatic SLE, 24 asymptomatic SLE	US	Hands, wrists	US-confirmed inflammation in 68%, but BILAG A or B met by only 38% and the SLEDAI musculoskeletal criterion by 32%. Subclinical synovitis in 27%. RF- and anti-CCP-positive patients excluded. Interpretation: the definitive quantification of bidirectional misclassification, with an occult rhupus subgroup excluded by design.
Corzo García et al. (2024) (77)	Spain	83 SLE, 24 controls	MRI (contrast-enhanced)	Hands, wrists	Radiocarpal-radioulnar the commonest synovitis site (45.1%), then intercarpal (43.9%). MRI synovitis in 51.6% of patients with arthralgia but no clinical arthritis. Interpretation: MRI reproduces the ultrasound estimate of occult inflammation using an entirely different physical principle.
Mahmoud et al. (2021) (73)	United Kingdom	133 SLE with inflammatory joint pain	US	Hands, wrists	Radiocarpal-radioulnar the commonest synovitis site (45.1%), then intercarpal (43.9%). MRI synovitis in 51.6% of patients with arthralgia but no clinical arthritis. Interpretation: MRI reproduces the ultrasound estimate of occult inflammation using an entirely different physical principle. Positive scan in 78, of whom only 53 (68%) had a swollen joint, while 20% of the 66 patients with a swollen joint had a negative scan. After excluding 32 patients with fibromyalgia, US-positive patients showed significantly greater improvement in early morning stiffness after intramuscular glucocorticoid. Interpretation: the only prospective demonstration that imaging-defined synovitis predicts treatment response, and that fibromyalgia conceals that relationship.
Fagni et al. (2023) (69)	Italy and Germany	106 SLE scanned from a cohort of 400	US	Entheses, joints	Radiocarpal-radioulnar the commonest synovitis site (45.1%), then intercarpal (43.9%). MRI synovitis in 51.6% of patients with arthralgia but no clinical arthritis. Interpretation: MRI reproduces the ultrasound estimate of occult inflammation using an entirely different physical principle. US-confirmed enthesitis in 31 of 106 (29.2%). Over a median follow-up exceeding ten years these patients developed renal involvement less often (22.6% versus 46.5%) and responded poorly to B cell depletion. Interpretation: imaging may resolve a phenotype with a distinct trajectory and a distinct treatment response, though the retrospective design limits inference.
Tang et al. (2025) (10)	International	16 US, eight MRI and one optical imaging study	Systematic review and meta-analysis	Hands, wrists	Radiocarpal-radioulnar the commonest synovitis site (45.1%), then intercarpal (43.9%). MRI synovitis in 51.6% of patients with arthralgia but no clinical arthritis. Interpretation: MRI reproduces the ultrasound estimate of occult inflammation using an entirely different physical principle. Pooled odds ratio for the association between joint symptoms and US synovitis 10.89 (95% CI 4.02 to 29.48), and tenosynovitis 5.93 (95% CI 1.99 to 17.72), falling to 5.00 (95% CI 1.11 to 22.60) when restricted to physician-confirmed arthritis. Interpretation: quantifies the added value of imaging over examination, and the weakening on restriction indicates that clinical assessment adds noise.
Orsolini et al. (2023) (114)	Italy	SLE with articular involvement treated with belimumab	US	Hands, wrists	Radiocarpal-radioulnar the commonest synovitis site (45.1%), then intercarpal (43.9%). MRI synovitis in 51.6% of patients with arthralgia but no clinical arthritis. Interpretation: MRI reproduces the ultrasound estimate of occult inflammation using an entirely different physical principle. US-detected non-radiographic erosions associated with response to belimumab, in a cohort excluding RF- and ACPA-positive patients and Jaccoud’s arthropathy. Interpretation: preliminary evidence that an imaging feature can select patients for a specific agent, which is the practical use to which an articular measure would be put.
Ceccarelli et al. (2025, 2026) (95, 96)	Italy	13 SLE treated with anifrolumab, and an earlier case series	US	Hands (MCP, PIP)	Radiocarpal-radioulnar the commonest synovitis site (45.1%), then intercarpal (43.9%). MRI synovitis in 51.6% of patients with arthralgia but no clinical arthritis. Interpretation: MRI reproduces the ultrasound estimate of occult inflammation using an entirely different physical principle. Significant reduction in US inflammatory scores by one month, continuing at three and six months, with 90% showing loss of power Doppler signal in all joints by six months. Interpretation: an imaging endpoint detected an articular effect where a clinical joint count in a phase 3 trial did not, though the cohorts are small and uncontrolled.

## 6 Management of joint disease in SLE

Three sets of recommendations govern contemporary practice, and they differ in whether the lupus joint is addressed as an entity in its own right. The 2025 American College of Rheumatology guideline for the treatment of SLE is the only one to carry an arthritis-specific recommendation, conditionally advising initial therapy with methotrexate, a mycophenolic acid analogue, or azathioprine for persistent or recurrent active SLE arthritis in patients already established on hydroxychloroquine, with a low threshold for adding or substituting belimumab or anifrolumab where the response is inadequate, in preference to initial biological therapy (83). The 2026 British Society for Rheumatology (BSR) guideline for the management of children, young people, and adults with SLE (84) and the 2023 update of the EULAR recommendations (85) instead reach the joint indirectly, through recommendations stratified by overall disease severity in which arthritis appears as an exemplar of mild or moderate activity rather than as a target of treatment in its own right.

The ACR recommendation is graded conditional on very low to low certainty evidence, and none of the three documents states how articular activity should be quantified or what magnitude of change would constitute an articular response. A guideline can therefore specify which drug should be started for lupus arthritis while remaining unable to specify what improvement on that drug would look like, and treatment decisions for the lupus joint continue to be made using instruments that were designed to measure something else.

Beyond the articular domain, the three documents are largely concordant on the foundations of treatment, and such differences as exist between them lie in emphasis rather than in substance. Hydroxychloroquine is recommended for all patients unless contraindicated, at a long-term target dose of 5 mg/kg/day of actual body weight (84, 85), and the ACR guideline treats it as the background therapy on which its articular recommendation is predicated (83). Glucocorticoids, including intra-articular injection, may be used as bridging therapy while longer-acting agents take effect, with long-term oral use avoided where possible and maintenance targeted at 5 mg/day prednisolone or less (84). The ACR panel makes the same provision explicit for the joint, stating that acute or recurrent episodes of inflammatory arthritis may be managed with a course of non-steroidal anti-inflammatory drugs or a limited course of oral glucocorticoid while long-term therapy is established (83), with non-steroidal agents used at the lowest effective dose for short periods and avoided altogether in the presence of lupus nephritis.

Methotrexate occupies a comparable position in the BSR and ACR documents, carrying the strongest evidence grade of any conventional agent for non-renal disease in the former, where it is recommended at grade 1B both for mild disease refractory to hydroxychloroquine and, alongside mycophenolate, azathioprine, or calcineurin inhibitors, for moderate disease without organ-threatening features (84), and it was also identified by the ACR panel as the agent that may reasonably be chosen first where arthritis dominates the clinical picture without substantial involvement of other organs (83). The trial evidence in lupus arthritis specifically is nonetheless modest, resting substantially on a study demonstrating reduction in arthritis and in glucocorticoid requirement in antimalarial-resistant disease (86), which is the reason an agent that has been used in this indication for three decades could be graded no higher than conditional on very low to low certainty evidence.

The BSR and EULAR documents both formalize a treat-to-target approach, with remission or the Lupus Low Disease Activity State (LLDAS) as the primary goal (84, 85, 87), a strategy whose implementation in articular disease is constrained directly by the absence of a valid articular target. A patient may satisfy the definition of low disease activity while retaining power Doppler-positive synovitis that no component of the target definition is capable of registering.

For patients with moderate-to-severe non-renal disease, or who cannot taper glucocorticoids despite immunosuppression, biological therapy is recommended. Belimumab is supported at grade 1A in autoantibody-positive patients with high disease activity, with rituximab or anifrolumab appropriate in selected presentations. Both EULAR and ACR guidelines name belimumab and anifrolumab as the preferred additions where conventional immunosuppression fails to control disease activity in the extra-renal domain (83–85).

The articular evidence for these agents is of variable quality, and belimumab has the most joint-specific real-world data, with improvement in pain index and in swollen and tender joint counts sustained to 24 months (88). In the BeRLiSS-JS cohort, DAS28 remission was reached in 46%, 57%, and 71% of patients at 6, 12, and 24 months, glucocorticoid discontinuation rose from 8.5% to 28.9% over the same period, and shorter disease duration and lower baseline DAS28 predicted remission, which suggests benefit from early use (89). Rituximab has consistent but small-scale evidence in rhupus, with DAS28 falling from 5.73 to 3.02 at 6 months in nine patients (90) and comparable improvements, including in ultrasound indices, in a six-patient longitudinal series (91). Tocilizumab produced complete resolution of arthritis in four of seven patients with active disease, but five of the seven experienced reactivation within 8 weeks of stopping treatment, indicating that IL-6 blockade suppresses rather than resets articular inflammation (92).

The observational and trial data on anifrolumab diverge, since uncontrolled series suggest substantial articular benefit, with musculoskeletal involvement falling from 63% at baseline to 8% at 6 months in one small cohort (93). In TULIP-2, however, among patients with at least six swollen and six tender joints at baseline, the proportions achieving a 50% or greater reduction in both counts did not differ significantly between anifrolumab and placebo (42.2% vs. 37.5%, difference 4.7%, 95% CI −13.5 to 17.6) (94). Read alongside the finding that interferon-high status does not distinguish patients by musculoskeletal involvement (17, 42), this negative articular result is more coherent than it first appears.

Imaging has since gone some way towards resolving this discrepancy. In a prospective study of 13 patients with SLE treated with anifrolumab for musculoskeletal involvement, ultrasound inflammatory scores fell significantly by 1 month and continued to fall at 3 and 6 months, with 90% of patients showing loss of power Doppler signal in all assessed joints by 6 months (95), findings anticipated by an earlier case series from the same group (96). These cohorts are small and uncontrolled, and the improvement in ultrasound scores cannot be separated from concurrent changes in background therapy. The direction of the finding is nonetheless informative, in that an imaging endpoint detected an articular effect in a setting where a swollen and tender joint count in a phase 3 trial did not, which is the outcome that would be predicted if clinical joint counts are the less sensitive instrument.

Janus kinase (JAK) inhibition has produced the most consistent joint signal among targeted agents, though the trial context requires care. In the phase 2 baricitinib trial, patients receiving 4 mg daily showed improvement in worst joint pain and in 28-tender joint count relative to placebo at week 24 (97). SLE-BRAVE-II also reported a difference in 28-tender joint count favoring baricitinib 4 mg (−0.91, 95% CI −1.74 to −0.07, p = 0.033), but that trial did not meet its primary endpoint of SLE Responder Index 4 (SRI-4) response, and the articular result must be interpreted as a secondary outcome within a negative trial (98). A retrospective study of tofacitinib in 22 patients with SLE and arthritis found significant reductions in 28-tender and 28-swollen joint counts at 24 weeks (99).

The limits of guideline-directed care are clearest in deforming disease, where the ACR voting panel was unable to reach consensus on either medical or surgical management of Jaccoud’s arthropathy and could offer only occupational therapy, physiotherapy, splinting, or bracing on the basis of panel experience (83). That absence of recommendation is appropriate given the evidence available, and it identifies precisely the phenotypes that the interferon-stratified data discussed above suggest are mechanistically distinct from the synovitis for which the available agents were developed, and for which an instrument sensitive to tendon and capsular rather than synovial pathology would be needed before the therapeutic question could properly be asked.

## 7 Emerging therapies and the articular endpoint gap

The therapeutic landscape in SLE has changed substantially, with four mechanistically distinct agents reaching or approaching registration. Examined specifically for what they report about the joints, they reveal that the measurement problem described above is not confined to the clinic.

### 7.1 Articular endpoints across recent trial program

Obinutuzumab, a type II anti-CD20 antibody producing deeper B cell depletion than rituximab, improved complete renal response in proliferative lupus nephritis in the phase 2 NOBILITY trial (100) and in the phase 3 REGENCY trial (101). In non-renal SLE, the ALLEGORY trial reported an SRI-4 response of 76.7% vs. 53.5% (adjusted difference 23.1 percentage points, 95% CI 12.5–33.6), among the largest effect sizes yet reported in a phase 3 SLE trial (102). Yet no particular outcome appears at any level of any of these trials. The key secondary hierarchy in ALLEGORY comprises BICLA, sustained glucocorticoid reduction, sustained SRI-4, SRI-6, and time to first BILAG flare. Currently there is no joint count, BILAG musculoskeletal domain endpoint, or SLEDAI arthritis descriptor endpoint; however, *post-hoc* analysis may be more forthcoming.

Telitacicept, a TACI-Fc fusion protein blocking both BLyS and APRIL, achieved a modified SRI-4 response of 67.1% vs. 32.7% at week 52 in a phase 3 trial of 335 patients (103). The articular data, however, exist only in the earlier phase 2b study, in which BILAG musculoskeletal domain improvement was seen in 78.8%–89.3% of patients across dose groups compared with 42.9% on placebo, with corresponding SLEDAI musculoskeletal improvements of 70.8%–80.0% vs. 34.5% (104). These are among the largest articular signals reported for any agent in SLE, yet they derive from subgroups of 22 to 36 patients, no joint counts were collected, and articular outcomes were absent entirely from the registrational trial. The evidence base moved backward on the joints as the program advanced.

Dapirolizumab pegol, an anti-CD40L Fab’, met its primary endpoint in the phase 3 PHOENYCS GO trial, with BICLA response in 50% versus 35% (difference 14.6 percentage points, 95% CI 3.3–25.8, p = 0.011) (105). Joint counts were declared secondary endpoints in the phase 2b study, but no results were reported in the primary publication (106).

Deucravacitinib, an allosteric TYK2 inhibitor, is the only agent among these to have had an articular outcome placed within a multiplicity-controlled testing hierarchy, the phase 2 PAISLEY trial having enriched for musculoskeletal or mucocutaneous involvement at entry and positioned joint counts among its formal secondary endpoints, albeit last in the testing sequence. The endpoint was met, although the magnitude of separation between the groups was small, with an adjusted mean change in 40-joint active joint count of −8.9 with deucravacitinib 3 mg twice daily versus −7.6 with placebo (p = 0.001), a between-group difference of 1.3 joints. The prespecified exploratory Joint Count-50 endpoint was achieved by 68.3% of patients on 3 mg twice daily versus 45.3% on placebo. The phase 3 trial is ongoing and results are currently awaited (107).

One program has already placed a joint count at the center of a lupus trial. The LILAC study of litifilimab, a monoclonal antibody directed against blood dendritic cell antigen 2 that suppresses type I interferon production by plasmacytoid dendritic cells, was redesigned during its conduct so that its primary endpoint became the change from baseline in total active joint count at week 24 in patients with SLE, arthritis, and active skin disease (108). Among the 102 participants analyzed, the least-squares mean change was −15.0 joints with litifilimab against −11.6 with placebo, a difference of 3.4 joints (95% CI −6.7 to −0.2, p = 0.04), although most secondary endpoints did not support the primary result. The trial demonstrates both that an articular primary endpoint is achievable in SLE and why it is difficult, since a placebo group improved by 11.6 joints from a baseline of 21.6, a magnitude of change that no plausible mechanism of drug action can plausibly explain.

Across these registrational or near-registrational trials, none have used ultrasound or MRI to define or measure articular response. Every articular claim in this literature rests on a clinician’s assessment, applied to a disease in which two imaging modalities have independently shown that clinical examination misses roughly half of objectively inflamed joints.

Trials that enroll on the basis of joint swelling assemble a population that is diluted by patients without articular inflammation, a fraction that the prevalence of concomitant fibromyalgia alone predicts will be substantial (18, 19), while simultaneously excluding patients whose subclinical synovitis places them among those most likely to respond. Placebo response on articular endpoints across this literature is correspondingly high, lying between 37.5% and 50.5% in every program that reported an articular outcome at all (94, 102, 105), and it follows arithmetically from a population constituted in this way, since patients whose joint symptoms have no inflammatory substrate can improve without any drug effect whatever. An agent with genuine articular efficacy could therefore fail on an endpoint of this kind, and nothing in the published literature permits a determination of whether that has already occurred.

The field has recognized the problem and has begun, within the past two years, to address it. The Treatment Response Measure for SLE initiative conducted a two-stage modified Delphi exercise among 87 clinicians and 13 patient representatives to establish which domains of disease activity a new trial outcome measure should contain, and arthritis was one of only eight domains from an initial 34 to satisfy consensus thresholds simultaneously on importance, appropriateness for evaluating change, representation among patients with active disease, and measurability within a randomized trial (TRM-SLE). That result establishes the articular domain as one the field considers both necessary and feasible to measure, although a Delphi exercise defines what should be captured rather than supplying the instrument with which to capture it (109).

### 7.2 Chimeric antigen receptor T cell therapies for lupus arthropathies

Autologous CD19-directed chimeric antigen receptor T cell therapy has produced drug-free remission in refractory SLE of a depth not previously achieved, first in a single patient and subsequently in a case series with extended follow-up in which all patients discontinued immunosuppression (110, 111). The mechanism is held to be an immune reprogramming achieved through depletion of B cells in tissue as well as in blood, which has now been demonstrated directly in serial lymph node tissue sampling (112), and compound constructs targeting both CD19 and B cell maturation antigen have entered early trials (113). However, no direct articular outcome has been reported from any of these studies beyond global disease activity indices, and the numbers treated remain small, so little can presently be said about the effect of these therapies on lupus arthritis specifically.

The same reasoning applies to the immune complex axis. Modulation of Fc gamma receptor signaling has reached clinical evaluation in SLE (47), and complement consumption is associated with ultrasound-detected joint abnormality (46), yet no complement-directed or Fc receptor-directed agent has been examined against an articular endpoint. The broader point that emerges from this section is that the therapeutic literature in lupus arthritis is organized by drug rather than by phenotype. The data reviewed above already suggest which pairings would be worth testing, in that IFN-associated non-deforming synovitis is the phenotype in which IFN-directed therapy should work, ultrasound-detected erosion has been reported to predict response to belimumab (114), ultrasound-detected enthesitis has been associated with failure of B cell depletion (69), and clinically irreducible deformity is the phenotype in which no currently available agent has a defensible rationale. None of these pairings can be tested without an articular outcome measure capable of distinguishing the phenotypes in the first place. **Table 4** provides an account of articular outcomes in response to emerging therapies in SLE.

**Table 4:** Articular outcomes across current and emerging therapies in systemic lupus erythematosus, classified by the status of the articular outcome within each trial program.

Agent and target	Key trial	Articular outcome reported	Status of that outcome	Imaging used
Litifilimab, BDCA2 on plasmacytoid dendritic cells	LILAC, phase 2, n = 102 analyzed	Change in total active joint count −15.0 vs. −11.6, difference 3.4 joints (95% CI −6.7 to −0.2, p = 0.04)	Primary endpoint	No
Anifrolumab, IFNAR1	TULIP-2, phase 3	At least 50% reduction in swollen and tender joint counts in 42.2% vs. 37.5% (difference 4.7%, 95% CI −13.5 to 17.6)	Prespecified subgroup analysis	Not in the trial, although separate prospective ultrasound cohorts have been reported
Deucravacitinib, TYK2	PAISLEY, phase 2, n = 363	Adjusted mean change in 40-joint active joint count −8.9 vs. −7.6 (p = 0.001), a difference of 1.3 joints	Multiplicity-controlled secondary endpoint, last in the testing hierarchy	No
Baricitinib, JAK1 and JAK2	SLE-BRAVE-II, phase 3	28-tender joint count difference -0.91 (95% CI −1.74 to −0.07, p = 0.033)	Secondary endpoint within a trial that did not meet its primary endpoint	No
Dapirolizumab pegol, CD40 ligand	PHOENYCS GO, phase 3	At least 50% reduction in combined tender and swollen joint count, reported only in conference abstract form	*Post-hoc*, absent from the primary publication	No
Telitacicept, BLyS and APRIL	Phase 2b, n = 249, and phase 3, n = 335	BILAG musculoskeletal domain improvement in 78.8% to 89.3% vs. 42.9% on placebo in phase 2b only	Organ-domain secondary in phase 2b, absent from the registrational trial	No
Obinutuzumab, CD20 type II	NOBILITY, REGENCY and ALLEGORY	None reported in any trial	Absent at every level	No
Belimumab, soluble BLyS	Observational cohorts including BeRLiSS-JS	Sustained improvement in swollen and tender joint counts, DAS28 remission in 46%, 57%, and 71% at 6, 12, and 24 months	Observational	Ultrasound erosion reported to predict response in one retrospective cohort
Rituximab, CD20 type I	Rhupus case series, n = 9 and n = 6	DAS28 5.73 to 3.02 at 6 months, with improvement in power Doppler indices	Uncontrolled	Ultrasound in one series
Tocilizumab, IL-6 receptor	Open-label phase I, n = 16	Complete resolution of arthritis in four of seven, with reactivation in five of seven within eight weeks of stopping	Exploratory	No
CD19 CAR T cells	Case series in refractory SLE	No articular outcome reported beyond global disease activity indices	Not assessed	No

Aside from separate observational ultrasound cohorts, no trial listed here used imaging to define or measure articular response.

## 8 Conclusions and future directions

Joint disease is the most prevalent manifestation of SLE and one of the least well measured. Ultrasound and MRI have each demonstrated, in independent cohorts, that objective synovial inflammation is present in approximately half of patients whose joint examination is unremarkable, and that erosive disease occurs in ACPA-negative patients who would not be classified as having rhupus. Recent large-cohort data indicate that lupus arthropathy may comprise more than one biological trajectory, separable at the point of assessment by IFN status, although whether these represent distinct entities or successive stages of a shared process is not yet established. Meanwhile, the immunopathology of the lupus synovium rests on two primary datasets, neither generated with single-cell methods, and the registrational trials of four new agents contain, between them, almost no interpretable articular data.

The lupus synovium requires modern interrogation, since single-cell transcriptomic and mass cytometric profiling of SLE synovial tissue has not been performed, and until it is, every cellular hypothesis about this compartment, including the roles of Tph and DN2 cells that are prominent elsewhere in systemic lupus biology, remains an extrapolation from rheumatoid arthritis.

The absence of single-cell profiling of lupus synovial tissue has understandable causes and naming them is necessary if the gap is to be closed. Large joint effusions are an uncommon presentation of lupus arthritis; the dominant lesion is tenosynovial rather than intra-articular, few centers offer synovial biopsy in lupus, and the yield of immune cells from small samples is low, so the tissue that transformed understanding of rheumatoid arthritis is not routinely obtainable in this disease. None of these obstacles is now insurmountable. Ultrasound-guided minimally invasive biopsy reliably obtains high-quality synovial tissue from small joints as well as large joints (115), and has been used to stratify treatment in a randomized trial in rheumatoid arthritis (116). Spatial transcriptomic methods preserve tissue architecture and require fewer cells than dissociation-based approaches; single-cell sequencing combined with surface protein profiling recovers phenotype where transcript counts are low, and paired analysis of blood and synovium would establish directly whether the populations expanded in the circulation in SLE are present in the joint. A multicenter biopsy program in lupus arthritis, modeled on those already established in rheumatoid arthritis, is the single intervention that would most change what is known about this compartment.

An SLE-specific imaging scoring system is also required, given that the recommendation to replace RAMRIS with a lupus-specific instrument was made in 2018 and has not yet been acted upon, while the equivalent work for ultrasound has not begun. An articular outcome measure combining imaging-defined inflammation with the serum and synovial biomarkers that track it should then be developed and validated for use in trials, and the derivation of a first candidate instrument indicates that this is achievable rather than merely desirable. Lupus arthritis lacks the precision now applied to lupus nephritis, which rests on tissue-based classification, validated activity indices, and accepted surrogate endpoints. The obstacles to providing the same for the joint are real, since the pathogenesis of lupus arthritis is incompletely understood and the tissue on which that understanding depends is difficult to obtain, but they are practical and methodological rather than conceptual, and each of them is now addressable with methods already in routine use elsewhere in rheumatology.

## References

[1] Zoma A . Musculoskeletal involvement in systemic lupus erythematosus. Lupus. (2004) 13:851–3. doi: 10.1191/0961203303lu2021oa 15580980

[2] Ball EM Bell AL . Lupus arthritis--do we have a clinically useful classification? Rheumatol (Oxford). (2012) 51:771–9. doi: 10.1093/rheumatology/ker381 22179731

[3] Kamen DL Birt JA Hadi MA Gibbons E Bushnell DM Yu R . Patient-reported disease impact of systemic lupus erythematosus with active joint symptoms: Results from the systemic lupus erythematosus-update survey. Lupus. (2023) 32:342–51. doi: 10.1177/09612033221147479 36541633

[4] Cornet A Andersen J Myllys K Edwards A Arnaud L . Living with systemic lupus erythematosus in 2020: a European patient survey. Lupus Sci Med. (2021) 8. doi: 10.1136/lupus-2020-000469 33849920 PMC8051432

[5] Aringer M Costenbader K Daikh D Brinks R Mosca M Ramsey-Goldman R . 2019 European League Against Rheumatism/American College of Rheumatology classification criteria for systemic lupus erythematosus. Ann Rheum Dis. (2019) 78:1151–9. doi: 10.1002/art.40930 31383717

[6] Gladman DD Ibañez D Urowitz MB . Systemic lupus erythematosus disease activity index 2000. J Rheumatol. (2002) 29:288–91. doi: 10.3899/jrheum.130030 11838846

[7] Murphy G Isenberg DA . SLE-associated arthropathy: truly a benign entity? Nat Rev Rheumatol. (2012) 8:695–6. doi: 10.1038/nrrheum.2012.200 23147902

[8] Ceccarelli F Perricone C Cipriano E Massaro L Natalucci F Capalbo G . Joint involvement in systemic lupus erythematosus: From pathogenesis to clinical assessment. Semin Arthritis Rheum. (2017) 47:53–64. doi: 10.1016/j.semarthrit.2017.03.022 28465078

[9] Shumilova A Vital EM . Musculoskeletal manifestations of systemic lupus erythematosus. Best Pract Res Clin Rheumatol. (2023) 37:101859. doi: 10.1016/j.berh.2023.101859 37620235

[10] Tang W Khalili L Gong R Souvignier M Wang X Murray S . Advanced imaging in the evaluation of lupus arthritis: A systematic literature review and meta-analysis. Semin Arthritis Rheum. (2025) 71:152661. doi: 10.2139/ssrn.4854881 39985848

[11] Di Matteo A Corzo P Filippucci E . The role of ultrasound and magnetic resonance imaging in the assessment of musculoskeletal involvement in systemic lupus erythematosus: A review of recent advances and insights. Best Pract Res Clin Rheumatol. (2025) 39:102042. doi: 10.1016/j.berh.2025.102042 40011120

[12] Grossman JM . Lupus arthritis. Best Pract Res Clin Rheumatol. (2009) 23:495–506. doi: 10.1016/j.berh.2009.04.003 19591780

[13] van Vugt RM Derksen RH Kater L Bijlsma JW . Deforming arthropathy or lupus and rhupus hands in systemic lupus erythematosus. Ann Rheum Dis. (1998) 57:540–4. doi: 10.1136/ard.57.9.540 9849313 PMC1752746

[14] Spronk PE ter Borg EJ Kallenberg CG . Patients with systemic lupus erythematosus and Jaccoud's arthropathy: a clinical subset with an increased C reactive protein response? Ann Rheum Dis. (1992) 51:358–61. doi: 10.1136/ard.51.3.358 1575582 PMC1004661

[15] Li J Wu H Huang X Xu D Zheng W Zhao Y . Clinical analysis of 56 patients with rhupus syndrome: manifestations and comparisons with systemic lupus erythematosus: a retrospective case-control study. Med (Baltimore). (2014) 93:e49. doi: 10.1097/md.0000000000000049 25170930 PMC4616332

[16] Benavente EP Paira SO . Rhupus: report of 4 cases. Reumatol Clin. (2011) 7:333–5. doi: 10.1016/j.reumae.2010.12.003 21925450

[17] Mehta P Kharouf F Carrizo-Abarza V Wither J Patel M Li Q . Characterizing arthritis subtypes in lupus: prevalence, clinical features, and role of type I interferon signatures. Rheumatol (Oxford). (2026) 65(8):1-9. doi: 10.1093/rheumatology/keag379 42484290

[18] Mistry S Daoud A Magrey MN Pamuk ON . The frequency of fibromyalgia in patients with systemic lupus erythematosus and associated factors: a systematic review and meta-analysis. Clin Rheumatol. (2025) 44:9–21. doi: 10.1007/s10067-024-07188-9 39424681

[19] Wolfe F Petri M Alarcón GS Goldman J Chakravarty EF Katz RS . Fibromyalgia, systemic lupus erythematosus (SLE), and evaluation of SLE activity. J Rheumatol. (2009) 36:82–8. doi: 10.3899/jrheum.080212 19004039 PMC2944223

[20] Nzeusseu Toukap A Galant C Theate I Maudoux AL Lories RJ Houssiau FA . Identification of distinct gene expression profiles in the synovium of patients with systemic lupus erythematosus. Arthritis Rheum. (2007) 56:1579–88. doi: 10.1002/art.22578 17469140

[21] Hubbard EL Catalina MD Heuer S Bachali P Robl R Geraci NS . Analysis of gene expression from systemic lupus erythematosus synovium reveals myeloid cell-driven pathogenesis of lupus arthritis. Sci Rep. (2020) 10:17361. doi: 10.1038/s41598-020-74391-4 33060686 PMC7562741

[22] Natour J Montezzo LC Moura LA Atra E . A study of synovial membrane of patients with systemic lupus erythematosus (SLE). Clin Exp Rheumatol. (1991) 9:221–5. 1879082

[23] Zhang F Wei K Slowikowski K Fonseka CY Rao DA Kelly S . Defining inflammatory cell states in rheumatoid arthritis joint synovial tissues by integrating single-cell transcriptomics and mass cytometry. Nat Immunol. (2019) 20:928–42. doi: 10.1038/s41590-019-0378-1 31061532 PMC6602051

[24] Zhang F Jonsson AH Nathan A Millard N Curtis M Xiao Q . Deconstruction of rheumatoid arthritis synovium defines inflammatory subtypes. Nature. (2023) 623:616–24. doi: 10.1038/s41586-023-06708-y 37938773 PMC10651487

[25] Harre U Georgess D Bang H Bozec A Axmann R Ossipova E . Induction of osteoclastogenesis and bone loss by human autoantibodies against citrullinated vimentin. J Clin Invest. (2012) 122:1791–802. doi: 10.1172/jci60975 22505457 PMC3336988

[26] Engdahl C Bang H Dietel K Lang SC Harre U Schett G . Periarticular bone loss in arthritis is induced by autoantibodies against citrullinated vimentin. J Bone Miner Res. (2017) 32:1681–91. doi: 10.1002/jbmr.3611 28425620

[27] McInnes IB Schett G . The pathogenesis of rheumatoid arthritis. N Engl J Med. (2011) 365:2205–19. doi: 10.1056/nejmra1004965 22150039

[28] Rao DA Gurish MF Marshall JL Slowikowski K Fonseka CY Liu Y . Pathologically expanded peripheral T helper cell subset drives B cells in rheumatoid arthritis. Nature. (2017) 542:110–4. doi: 10.1038/nature20810 28150777 PMC5349321

[29] Bocharnikov AV Keegan J Wacleche VS Cao Y Fonseka CY Wang G . PD-1hiCXCR5- T peripheral helper cells promote B cell responses in lupus via MAF and IL-21. JCI Insight. (2019) 4. doi: 10.1172/jci.insight.130062 31536480 PMC6824311

[30] Makiyama A Chiba A Noto D Murayama G Yamaji K Tamura N . Expanded circulating peripheral helper T cells in systemic lupus erythematosus: association with disease activity and B cell differentiation. Rheumatol (Oxford). (2019) 58:1861–9. doi: 10.1093/rheumatology/kez077 30879065

[31] Jenks SA Cashman KS Zumaquero E Marigorta UM Patel AV Wang X . Distinct effector B cells induced by unregulated toll-like receptor 7 contribute to pathogenic responses in systemic lupus erythematosus. Immunity. (2018) 49:725–739.e6. doi: 10.1016/j.immuni.2018.08.015 30314758 PMC6217820

[32] Wing E Sutherland C Miles K Gray D Goodyear CS Otto TD . Double-negative-2 B cells are the major synovial plasma cell precursor in rheumatoid arthritis. Front Immunol. (2023) 14:1241474. doi: 10.3389/fimmu.2023.1241474 37638026 PMC10450142

[33] Croft AP Campos J Jansen K Turner JD Marshall J Attar M . Distinct fibroblast subsets drive inflammation and damage in arthritis. Nature. (2019) 570:246–51. doi: 10.1038/s41586-019-1263-7 31142839 PMC6690841

[34] Wei K Korsunsky I Marshall JL Gao A Watts GFM Major T . Notch signalling drives synovial fibroblast identity and arthritis pathology. Nature. (2020) 582:259–64. doi: 10.1038/s41586-020-2222-z 32499639 PMC7841716

[35] Kayakabe K Kuroiwa T Sakurai N Ikeuchi H Kadiombo AT Sakairi T . Interleukin-6 promotes destabilized angiogenesis by modulating angiopoietin expression in rheumatoid arthritis. Rheumatol (Oxford). (2012) 51:1571–9. doi: 10.1093/rheumatology/kes093 22596210

[36] Baillet A Gossec L Paternotte S Etcheto A Combe B Meyer O . Evaluation of serum interleukin-6 level as a surrogate marker of synovial inflammation and as a factor of structural progression in early rheumatoid arthritis: results from a French national multicenter cohort. Arthritis Care Res (Hoboken). (2015) 67:905–12. doi: 10.1002/acr.22513 25384758

[37] Sippl N Faustini F Rönnelid J Turcinov S Chemin K Gunnarsson I . Arthritis in systemic lupus erythematosus is characterized by local IL-17A and IL-6 expression in synovial fluid. Clin Exp Immunol. (2021) 205:44–52. doi: 10.1111/cei.13585 33576004 PMC8209560

[38] Vincent FB Northcott M Hoi A Mackay F Morand EF . Clinical associations of serum interleukin-17 in systemic lupus erythematosus. Arthritis Res Ther. (2013) 15:R97. doi: 10.1186/ar4277 23968496 PMC3979031

[39] Crispín JC Oukka M Bayliss G Cohen RA Van Beek CA Stillman IE . Expanded double negative T cells in patients with systemic lupus erythematosus produce IL-17 and infiltrate the kidneys. J Immunol. (2008) 181:8761–6. doi: 10.4049/jimmunol.181.12.8761 PMC2596652 19050297

[40] Baechler EC Batliwalla FM Karypis G Gaffney PM Ortmann WA Espe KJ . Interferon-inducible gene expression signature in peripheral blood cells of patients with severe lupus. Proc Natl Acad Sci USA. (2003) 100:2610–5. doi: 10.1073/pnas.0337679100 12604793 PMC151388

[41] Smith JD Garcia LW Bonilla D Li Q Keeling S Avina-Zubieta JA . Type I Interferon status and clinical manifestations in a large cohort of patients with systemic lupus erythematosus. Joint Bone Spine. (2026) 93:106081. doi: 10.3899/jrheum.2025-0314.108 42202901

[42] Arnaud L Furie R Morand EF Aringer M Peschken C Desta B . Burden of systemic lupus erythematosus in clinical practice: baseline data from the SLE Prospective Observational Cohort Study (SPOCS) by interferon gene signature. Lupus Sci Med. (2023) 10. doi: 10.1136/lupus-2023-001032 38123459 PMC10749026

[43] Miyachi K Iwamoto T Kojima S Ida T Suzuki J Yamamoto T . Relationship of systemic type I interferon activity with clinical phenotypes, disease activity, and damage accrual in systemic lupus erythematosus in treatment-naive patients: a retrospective longitudinal analysis. Arthritis Res Ther. (2023) 25:26. doi: 10.1186/s13075-023-03010-0 36803843 PMC9936752

[44] Souyris M Cenac C Azar P Daviaud D Canivet A Grunenwald S . TLR7 escapes X chromosome inactivation in immune cells. Sci Immunol. (2018) 3. doi: 10.1126/sciimmunol.aap8855 29374079

[45] Lood C Blanco LP Purmalek MM Carmona-Rivera C De Ravin SS Smith CK . Neutrophil extracellular traps enriched in oxidized mitochondrial DNA are interferogenic and contribute to lupus-like disease. Nat Med. (2016) 22:146–53. doi: 10.1038/nm.4027 26779811 PMC4742415

[46] Gabba A Piga M Vacca A Porru G Garau P Cauli A . Joint and tendon involvement in systemic lupus erythematosus: an ultrasound study of hands and wrists in 108 patients. Rheumatol (Oxford). (2012) 51:2278–85. doi: 10.1093/rheumatology/kes226 22956550

[47] Merrill JT Guthridge J Smith M June J Koumpouras F Machua W . Obexelimab in systemic lupus erythematosus with exploration of response based on gene pathway co-expression patterns: A double-blind, randomized, placebo-controlled, phase 2 trial. Arthritis Rheumatol. (2023) 75:2185–94. doi: 10.1002/art.42652 37459248

[48] Alivernini S MacDonald L Elmesmari A Finlay S Tolusso B Gigante MR . Distinct synovial tissue macrophage subsets regulate inflammation and remission in rheumatoid arthritis. Nat Med. (2020) 26:1295–306. doi: 10.1038/s41591-020-0939-8 32601335

[49] Arazi A Rao DA Berthier CC Davidson A Liu Y Hoover PJ . The immune cell landscape in kidneys of patients with lupus nephritis. Nat Immunol. (2019) 20:902–14. doi: 10.1038/s41590-019-0398-x 31209404 PMC6726437

[50] Damián-Abrego GN Cabiedes J Cabral AR . Anti-citrullinated peptide antibodies in lupus patients with or without deforming arthropathy. Lupus. (2008) 17:300–4. doi: 10.1177/0961203307087613 18413411

[51] Qing YF Zhang QB Zhou JG Yuan GH Wei J Xing Y . The detecting and clinical value of anti-cyclic citrullinated peptide antibodies in patients with systemic lupus erythematosus. Lupus. (2009) 18:713–7. doi: 10.1177/0961203309102817 19502267

[52] Massaro L Ceccarelli F Colasanti T Pendolino M Perricone C Cipriano E . Anti-carbamylated protein antibodies in systemic lupus erythematosus patients with articular involvement. Lupus. (2018) 27:105–11. doi: 10.1177/0961203317713141 28592200

[53] Ziegelasch M van Delft MA Wallin P Skogh T Magro-Checa C Steup-Beekman GM . Antibodies against carbamylated proteins and cyclic citrullinated peptides in systemic lupus erythematosus: results from two well-defined European cohorts. Arthritis Res Ther. (2016) 18:289. doi: 10.1186/s13075-016-1192-x 27912793 PMC5135817

[54] Eilertsen G Nikolaisen C Becker-Merok A Nossent JC . Interleukin-6 promotes arthritis and joint deformation in patients with systemic lupus erythematosus. Lupus. (2011) 20:607–13. doi: 10.1177/0961203310392432 21422077

[55] Ball EM Gibson DS Bell AL Rooney MR . Plasma IL-6 levels correlate with clinical and ultrasound measures of arthritis in patients with systemic lupus erythematosus. Lupus. (2014) 23:46–56. doi: 10.1177/0961203313512882 24243775

[56] Shahin AA Shaker OG Kamal N Hafez HA Gaber W Shahin HA . Circulating interleukin-6, soluble interleukin-2 receptors, tumor necrosis factor alpha, and interleukin-10 levels in juvenile chronic arthritis: correlations with soft tissue vascularity assessed by power Doppler sonography. Rheumatol Int. (2002) 22:84–8. doi: 10.1007/s00296-002-0191-1 12070682

[57] Kitchen J Kane D . Greyscale and power Doppler ultrasonographic evaluation of normal synovial joints: correlation with pro- and anti-inflammatory cytokines and angiogenic factors. Rheumatol (Oxford). (2015) 54:458–62. doi: 10.1093/rheumatology/keu354 25193808

[58] Ribeiro DS Lins CF Galvão V Santos WGD Rosa G Machicado V . Association of CXCL13 serum level and ultrasonographic findings of joints in patients with systemic lupus erythematosus and Jaccoud's arthropathy. Lupus. (2018) 27:939–46. doi: 10.1177/0961203317753557 29338586

[59] Schumacher HR Howe HS . Synovial fluid cells in systemic lupus erythematosus: light and electron microscopic studies. Lupus. (1995) 4:353–64. doi: 10.1177/096120339500400505 8563729

[60] Labowitz R Schumacher HR . Articular manifestations of systemic lupus erythematosus. Ann Intern Med. (1971) 74:911–21. doi: 10.7326/0003-4819-74-6-911 4931742

[61] Kane D Grassi W Sturrock R Balint PV . Musculoskeletal ultrasound--a state of the art review in rheumatology. Part 2: Clinical indications for musculoskeletal ultrasound in rheumatology. Rheumatol (Oxford). (2004) 43:829–38. doi: 10.1093/rheumatology/keh215 15161981

[62] Wright S Filippucci E Grassi W Grey A Bell A . Hand arthritis in systemic lupus erythematosus: an ultrasound pictorial essay. Lupus. (2006) 15:501–6. doi: 10.1191/0961203306lu2340oa 16942002

[63] Wakefield RJ Balint PV Szkudlarek M Filippucci E Backhaus M D'Agostino MA . Musculoskeletal ultrasound including definitions for ultrasonographic pathology. J Rheumatol. (2005) 32:2485–7. doi: 10.1016/b978-0-323-06551-1.00040-3 16331793

[64] Zayat AS Md Yusof MY Wakefield RJ Conaghan PG Emery P Vital EM . The role of ultrasound in assessing musculoskeletal symptoms of systemic lupus erythematosus: a systematic literature review. Rheumatol (Oxford). (2016) 55:485–94. doi: 10.1093/rheumatology/kev343 26447163 PMC5854027

[65] Qi W Yuen SY Popescu ML Yang R Giguère CE . The burden of enthesitis, inflammatory arthritis, and tenosynovitis on musculoskeletal ultrasound in systemic lupus erythematosus: a scoping review. J Rheumatol. (2026) 53:484–94. doi: 10.3899/jrheum.2025-0684 41241389

[66] Iagnocco A Ossandon A Coari G Conti F Priori R Alessandri C . Wrist joint involvement in systemic lupus erythematosus. An ultrasonographic study. Clin Exp Rheumatol. (2004) 22:621–4. 15485017

[67] Iagnocco A Ceccarelli F Rizzo C Truglia S Massaro L Spinelli FR . Ultrasound evaluation of hand, wrist and foot joint synovitis in systemic lupus erythematosus. Rheumatol (Oxford). (2014) 53:465–72. doi: 10.1093/rheumatology/ket376 24231444

[68] Ossandon A Iagnocco A Alessandri C Priori R Conti F Valesini G . Ultrasonographic depiction of knee joint alterations in systemic lupus erythematosus. Clin Exp Rheumatol. (2009) 27:329–32. 19473577

[69] Fagni F Bettiol A Silvestri E Fedi R Palermo A Urban ML . Prevalence and clinical associations of ultrasound-confirmed enthesitis in systemic lupus erythematosus. Rheumatol (Oxford). (2023) 62:3619–26. doi: 10.1093/rheumatology/kead123 36929914

[70] Yoon HS Kim KJ Baek IW Park YJ Kim WU Yoon CH . Ultrasonography is useful to detect subclinical synovitis in SLE patients without musculoskeletal involvement before symptoms appear. Clin Rheumatol. (2014) 33:341–8. doi: 10.1007/s10067-014-2502-9 24478125

[71] Dreyer L Jacobsen S Juul L Terslev L . Ultrasonographic abnormalities and inter-reader reliability in Danish patients with systemic lupus erythematosus - a comparison with clinical examination of wrist and metacarpophalangeal joints. Lupus. (2015) 24:712–9. doi: 10.1177/0961203314561666 25467390

[72] Zayat AS Mahmoud K Md Yusof MY Mukherjee S D'Agostino MA Hensor EMA . Defining inflammatory musculoskeletal manifestations in systemic lupus erythematosus. Rheumatol (Oxford). (2019) 58:304–12. doi: 10.1093/rheumatology/key277 30265343

[73] Mahmoud K Zayat AS Yusof MYM Dutton K Teh LS Yee CS . Ultrasound to identify systemic lupus erythematosus patients with musculoskeletal symptoms who respond best to therapy: the US Evaluation For mUsculoskeletal Lupus longitudinal multicentre study. Rheumatol (Oxford). (2021) 60:5194–204. doi: 10.1093/rheumatology/keab288 33792659 PMC8566203

[74] Ceccarelli F Perricone C Cipriano E Massaro L Natalucci F Spinelli FR . Usefulness of composite indices in the assessment of joint involvement in systemic lupus erythematosus patients: correlation with ultrasonographic score. Lupus. (2019) 28:383–8. doi: 10.1177/0961203319828527 30744521

[75] Tamai M Kawakami A Uetani M Fukushima A Arima K Fujikawa K . Magnetic resonance imaging (MRI) detection of synovitis and bone lesions of the wrists and finger joints in early-stage rheumatoid arthritis: comparison of the accuracy of plain MRI-based findings and gadolinium-diethylenetriamine pentaacetic acid-enhanced MRI-based findings. Mod Rheumatol. (2012) 22:654–8. doi: 10.3109/s10165-011-0575-8 22203216

[76] Navalho M Resende C Rodrigues AM Pereira da Silva JA Fonseca JE Campos J . Bilateral evaluation of the hand and wrist in untreated early inflammatory arthritis: a comparative study of ultrasonography and magnetic resonance imaging. J Rheumatol. (2013) 40:1282–92. doi: 10.3899/jrheum.120713 23729806

[77] Corzo Garcia P Garcia-Duitama I Agustí Claramunt A Duran Jordà X Monfort J Salman-Monte TC . Musculoskeletal involvement in systemic lupus erythematosus: a contrast-enhanced magnetic resonance imaging study in 107 subjects. Rheumatol (Oxford). (2024) 63:423–9. doi: 10.1093/rheumatology/kead223 37208172

[78] Ostendorf B Scherer A Specker C Mödder U Schneider M . Jaccoud's arthropathy in systemic lupus erythematosus: differentiation of deforming and erosive patterns by magnetic resonance imaging. Arthritis Rheum. (2003) 48:157–65. doi: 10.1002/art.10753 12528115

[79] Østergaard M Edmonds J McQueen F Peterfy C Lassere M Ejbjerg B . An introduction to the EULAR-OMERACT rheumatoid arthritis MRI reference image atlas. Ann Rheum Dis. (2005) 64 Suppl 1:i3–7. doi: 10.1057/9781137398758.0004 PMC1766828 15647420

[80] Haavardsholm EA Østergaard M Ejbjerg BJ Kvan NP Kvien TK . Introduction of a novel magnetic resonance imaging tenosynovitis score for rheumatoid arthritis: reliability in a multireader longitudinal study. Ann Rheum Dis. (2007) 66:1216–20. doi: 10.1136/ard.2006.068361 17392347 PMC1955157

[81] Zollars ES Hyer M Wolf B Chapin R . Measuring lupus arthritis activity using contrasted high-field MRI. Associations with clinical measures of disease activity and novel patterns of disease. Lupus Sci Med. (2018) 5:e000264. doi: 10.1136/lupus-2018-000264 30094039 PMC6069922

[82] Ball EM Tan AL Fukuba E McGonagle D Grey A Steiner G . A study of erosive phenotypes in lupus arthritis using magnetic resonance imaging and anti-citrullinated protein antibody, anti-RA33 and RF autoantibody status. Rheumatol (Oxford). (2014) 53:1835–43. doi: 10.1093/rheumatology/keu215 24850876

[83] Sammaritano LR Askanase A Bermas BL Dall'Era M Duarte-García A Hiraki LT . 2025 american college of rheumatology (ACR) guideline for the treatment of systemic lupus erythematosus. Arthritis Rheumatol. (2026) 78:1361–86. doi: 10.1001/jama.2024.2315 41187097 PMC13711245

[84] Md Yusof MY Smith EMD Lythgoe H Psarras A Armon K Beresford MW . The 2026 British Society for Rheumatology guideline for the management of children, young people and adults with systemic lupus erythematosus. Rheumatol (Oxford). (2026) 65. doi: 10.1093/rheumatology/keag223 42336388 PMC13290301

[85] Fanouriakis A Kostopoulou M Andersen J Aringer M Arnaud L Bae SC . EULAR recommendations for the management of systemic lupus erythematosus: 2023 update. Ann Rheum Dis. (2024) 83:15–29. doi: 10.1136/ard-2023-224762 37827694

[86] Rahman P Humphrey-Murto S Gladman DD Urowitz MB . Efficacy and tolerability of methotrexate in antimalarial resistant lupus arthritis. J Rheumatol. (1998) 25:243–6. 9489814

[87] Franklyn K Lau CS Navarra SV Louthrenoo W Lateef A Hamijoyo L . Definition and initial validation of a Lupus Low Disease Activity State (LLDAS). Ann Rheum Dis. (2016) 75:1615–21. doi: 10.1136/annrheumdis-2015-207726 26458737

[88] Germain G Worley K MacKnight SD Rubin B Bell CF Laliberté F . Evaluating the real-world effectiveness of belimumab in patients with SLE using SLE-related laboratory values and rheumatoid arthritis-derived disease activity measures: RAPID3, swollen joint count and tender joint count. Lupus Sci Med. (2024) 11. doi: 10.1136/lupus-2023-001111 38575172 PMC11002435

[89] Zen M Gatto M Depascale R Regola F Fredi M Andreoli L . Early and late response and glucocorticoid-sparing effect of belimumab in patients with systemic lupus erythematosus with joint and skin manifestations: Results from the Belimumab in Real Life Setting Study-Joint and Skin (BeRLiSS-JS). J Pers Med. (2023) 13. doi: 10.3390/jpm13040691 37109077 PMC10146447

[90] Andrade-Ortega L Irazoque-Palazuelos F Muñóz-López S Rosales-Don Pablo VM . Efficacy and tolerability of rituximab in patients with rhupus. Reumatol Clin. (2013) 9:201–5. doi: 10.1016/j.reumae.2013.03.004 23415605

[91] Piga M Gabba A Cauli A Garau P Vacca A Mathieu A . Rituximab treatment for 'rhupus syndrome': clinical and power-Doppler ultrasonographic monitoring of response. A longitudinal pilot study. Lupus. (2013) 22:624–8. doi: 10.1177/0961203313482741 23559669

[92] Illei GG Shirota Y Yarboro CH Daruwalla J Tackey E Takada K . Tocilizumab in systemic lupus erythematosus: data on safety, preliminary efficacy, and impact on circulating plasma cells from an open-label phase I dosage-escalation study. Arthritis Rheum. (2010) 62:542–52. doi: 10.1002/art.27221 20112381 PMC3057537

[93] Troldborg A Remkus L Eek D Deleuran B . Anifrolumab treatment improves patient-reported quality of life and decreases disease activity and corticosteroid use in patients with systemic lupus erythematosus: A qualitative study in Denmark. Lupus. (2024) 33:962–73. doi: 10.1177/09612033241261746 38901042 PMC11348625

[94] Morand EF Furie R Tanaka Y Bruce IN Askanase AD Richez C . Trial of anifrolumab in active systemic lupus erythematosus. N Engl J Med. (2020) 382:211–21. doi: 10.1056/nejmoa1912196 31851795

[95] Ceccarelli F Ciancarella C Moretti V Iaria D Spinelli FR Alessandri C . Anifrolumab induces a fast improvement in ultrasound-detected synovitis in systemic lupus erythematosus: Results from a 6-months follow-up ultrasound study. Lupus. (2026) 35(11):9612033261469032. doi: 10.1177/09612033261469032 42421189

[96] Ceccarelli F Ciancarella C Natalucci F Scorda A Tripodi G Garufi C . Rapid response to anifrolumab in lupus related joint involvement as assessed by ultrasonography: A case series study. Joint Bone Spine. (2025) 92:105830. doi: 10.1016/j.jbspin.2024.105830 39716637

[97] Wallace DJ Furie RA Tanaka Y Kalunian KC Mosca M Petri MA . Baricitinib for systemic lupus erythematosus: a double-blind, randomised, placebo-controlled, phase 2 trial. Lancet. (2018) 392:222–31. doi: 10.1016/s0140-6736(18)31363-1 30043749

[98] Petri M Bruce IN Dörner T Tanaka Y Morand EF Kalunian KC . Baricitinib for systemic lupus erythematosus: a double-blind, randomised, placebo-controlled, phase 3 trial (SLE-BRAVE-II). Lancet. (2023) 401:1011–9. doi: 10.1016/s0140-6736(22)02546-6 36848919

[99] Yan Q Liu J Long X Wu C Lin D Wu Y . Tofacitinib therapy in systemic lupus erythematosus with arthritis: a retrospective study. Clin Rheumatol. (2024) 43:3139–45. doi: 10.1007/s10067-024-07103-2 39136836

[100] Furie RA Aroca G Cascino MD Garg JP Rovin BH Alvarez A . B-cell depletion with obinutuzumab for the treatment of proliferative lupus nephritis: a randomised, double-blind, placebo-controlled trial. Ann Rheum Dis. (2022) 81:100–7. doi: 10.1136/annrheumdis-2021-220920 34615636 PMC8762029

[101] Furie RA Rovin BH Garg JP Santiago MB Aroca-Martínez G Zuta Santillán AE . Efficacy and safety of obinutuzumab in active lupus nephritis. N Engl J Med. (2025) 392:1471–83. doi: 10.1056/nejmoa2410965 39927615

[102] Furie RA Dall'Era M Vital EM Garg JP Irazoque Palazuelos F Zuta Santillán AE . Efficacy and safety of obinutuzumab in active systemic lupus erythematosus. N Engl J Med. (2026) 395:243–54. doi: 10.1056/nejmoa2516150 41789864

[103] van Vollenhoven RF Wang L Merrill JT Liu Y Bao C Li F . A phase 3 trial of telitacicept for systemic lupus erythematosus. N Engl J Med. (2025) 393:1475–85. doi: 10.1056/nejmoa2414719 41092329

[104] Wu D Li J Xu D Merrill JT van Vollenhoven RF Liu Y . Telitacicept in patients with active systemic lupus erythematosus: results of a phase 2b, randomised, double-blind, placebo-controlled trial. Ann Rheum Dis. (2024) 83:475–87. doi: 10.1136/ard-2023-224854 38129117 PMC10958275

[105] Clowse MEB Isenberg DA Merrill JT Dörner T Petri M Vital EM . Efficacy and safety of the CD40 ligand inhibitor dapirolizumab pegol in systemic lupus erythematosus (PHOENYCS GO): a randomised, double-blind, placebo-controlled, phase 3 trial. Lancet. (2026) 407:2291–304. doi: 10.1016/s0140-6736(26)00691-4 42214397

[106] Furie RA Bruce IN Dörner T Leon MG Leszczyński P Urowitz M . Phase 2, randomized, placebo-controlled trial of dapirolizumab pegol in patients with moderate-to-severe active systemic lupus erythematosus. Rheumatol (Oxford). (2021) 60:5397–407. doi: 10.1093/rheumatology/keab381 33956056 PMC9194804

[107] Morand E Pike M Merrill JT van Vollenhoven R Werth VP Hobar C . Deucravacitinib, a tyrosine kinase 2 inhibitor, in systemic lupus erythematosus: a phase II, randomized, double-blind, placebo-controlled trial. Arthritis Rheumatol. (2023) 75:242–52. doi: 10.1002/art.42391 PMC10100399 36369798

[108] Furie RA van Vollenhoven RF Kalunian K Navarra S Romero-Diaz J Werth VP . Trial of anti-BDCA2 antibody litifilimab for systemic lupus erythematosus. N Engl J Med. (2022) 387:894–904. doi: 10.1056/nejmoa2118025 36069871

[109] Connelly K Koelmeyer R Ayton D May J Gregory K Eades LE . Domains for inclusion in a novel treatment response measure for systemic lupus erythematosus (TRM-SLE): results of a modified Delphi study. Lupus Sci Med. (2025) 12. doi: 10.1136/lupus-2024-001484 40328675 PMC12067843

[110] Mackensen A Müller F Mougiakakos D Böltz S Wilhelm A Aigner M . Anti-CD19 CAR T cell therapy for refractory systemic lupus erythematosus. Nat Med. (2022) 28:2124–32. doi: 10.1038/s41591-022-02017-5 36109639

[111] Müller F Taubmann J Bucci L Wilhelm A Bergmann C Völkl S . CD19 CAR T-cell therapy in autoimmune disease - a case series with follow-up. N Engl J Med. (2024) 390:687–700. doi: 10.1056/NEJMoa2308917 38381673

[112] Tur C Eckstein M Bucci L Auth J Bergmann C Rauber S . Effects of different B-cell-depleting strategies on the lymphatic tissue. Ann Rheum Dis. (2025) 84:2065–74. doi: 10.1016/j.ard.2025.06.2120 40651933

[113] Wang W He S Zhang W Zhang H DeStefano VM Wada M . BCMA-CD19 compound CAR T cells for systemic lupus erythematosus: a phase 1 open-label clinical trial. Ann Rheum Dis. (2024) 83:1304–14. doi: 10.1136/ard-2024-225785 38777376

[114] Orsolini G Mastropaolo F Favaro E Piccinelli A Bertelle D Viapiana O . Ultrasonographic non-radiographic erosions could predict the efficacy of belimumab in articular systemic lupus erythematosus. Clin Rheumatol. (2023) 42:3083–8. doi: 10.1007/s10067-023-06635-3 37195373

[115] Kelly S Humby F Filer A Ng N Di Cicco M Hands RE . Ultrasound-guided synovial biopsy: a safe, well-tolerated and reliable technique for obtaining high-quality synovial tissue from both large and small joints in early arthritis patients. Ann Rheum Dis. (2015) 74:611–7. doi: 10.1136/annrheumdis-2013-204603 24336336

[116] Humby F Durez P Buch MH Lewis MJ Rizvi H Rivellese F . Rituximab versus tocilizumab in anti-TNF inadequate responder patients with rheumatoid arthritis (R4RA): 16-week outcomes of a stratified, biopsy-driven, multicentre, open-label, phase 4 randomised controlled trial. Lancet. (2021) 397:305–17. doi: 10.1016/s0140-6736(20)32341-2 33485455 PMC7829614

